# Influence of Recombination and GC-biased Gene Conversion on the Adaptive and Nonadaptive Substitution Rate in Mammals versus Birds

**DOI:** 10.1093/molbev/msy243

**Published:** 2018-12-27

**Authors:** Marjolaine Rousselle, Alexandre Laverré, Emeric Figuet, Benoit Nabholz, Nicolas Galtier

**Affiliations:** ISEM, Université de Montpellier, CNRS, IRD, EPHE, Montpellier, France

**Keywords:** GC-biased gene conversion, primates, birds, adaptive substitution rate, recombination, coding sequence evolution

## Abstract

Recombination is expected to affect functional sequence evolution in several ways. On the one hand, recombination is thought to improve the efficiency of multilocus selection by dissipating linkage disequilibrium. On the other hand, natural selection can be counteracted by recombination-associated transmission distorters such as GC-biased gene conversion (gBGC), which tends to promote G and C alleles irrespective of their fitness effect in high-recombining regions. It has been suggested that gBGC might impact coding sequence evolution in vertebrates, and particularly the ratio of nonsynonymous to synonymous substitution rates (dN/dS). However, distinctive gBGC patterns have been reported in mammals and birds, maybe reflecting the documented contrasts in evolutionary dynamics of recombination rate between these two taxa. Here, we explore how recombination and gBGC affect coding sequence evolution in mammals and birds by analyzing proteome-wide data in six species of Galloanserae (fowls) and six species of catarrhine primates. We estimated the dN/dS ratio and rates of adaptive and nonadaptive evolution in bins of genes of increasing recombination rate, separately analyzing AT → GC, GC → AT, and G ↔ C/A ↔ T mutations. We show that in both taxa, recombination and gBGC entail a decrease in dN/dS. Our analysis indicates that recombination enhances the efficiency of purifying selection by lowering Hill–Robertson effects, whereas gBGC leads to an overestimation of the adaptive rate of AT → GC mutations. Finally, we report a mutagenic effect of recombination, which is independent of gBGC.

## Introduction

Understanding the relative importance of natural selection versus nonadaptive forces is a central question in molecular evolution (Kimura 1983; Gillespie 1994). Over the past few years, a number of methods and statistics have been developed to assess the efficacy of positive and purifying selection.

Many of these methods are based on the comparison of the nonsynonymous and synonymous mutation and substitution rates. Nonsynonymous changes are supposedly under selective effects, whereas synonymous mutations are used as a control for nonselective processes. Statistics commonly used to estimate the extent of selective pressure acting at the sequence level include dN/dS, the ratio of nonsynonymous over synonymous substitution rate (between species), and *π*_n_/*π*_s_, the ratio of nonsynonymous over synonymous nucleotide diversity (within species). Combining divergence and polymorphism data can provide a way to disentangle adaptive from nonadaptive effects (McDonald and Kreitman 1991) and to estimate the proportion of amino acid substitutions that resulted from positive selection—a proportion called *α* (Smith and Eyre-Walker 2002). The most recent versions of this approach, grouped under the name “DFE-*α*” (Eyre-Walker et al. 2006; Keightley and Eyre-Walker 2007; Eyre-Walker and Keightley 2009; Galtier 2016; Tataru et al. 2017), extract information on the distribution of the fitness effects (DFE) of nonsynonymous mutations from the joint analysis of the synonymous and nonsynonymous site frequency spectra (SFS). The expected dN/dS under near neutrality is deduced from the analysis of polymorphism data, and the difference between observed and expected dN/dS provides estimates for *α* and for the per synonymous substitution rate of adaptive and nonadaptive amino-acid substitution, respectively *ω*_a_ = *α*(dN/dS) and *ω*_na_ = (1 – *α*)(dN/dS).

The methods reviewed above rely on the assumption that only drift and mutation determine the synonymous component, whereas drift, mutation, and selection determine the nonsynonymous component. However, coding sequences may be affected by other forces, such as selection on codon usage and GC-biased gene conversion (gBGC), which can modify the expectations regarding the dN/dS ratio, the *π*_n_/*π*_s_ ratio and the DFE-*α* method (Galtier et al. 2009; Berglund et al. 2009; Ratnakumar et al. 2010; Bolívar et al. 2016; Corcoran et al. 2017). Here, we focus on gBGC as a potential source of bias in the estimation of the rate of adaptive and nonadaptive amino acid substitution.

gBGC originates from a repair bias during meiotic recombination that results in a distorted segregation favoring G and C over A and T alleles in highly recombining regions (Eyre-Walker 1993; Galtier et al. 2001; Glémin et al. 2015). A large body of literature provides evidence for gBGC in a wide range of organisms (Eyre-Walker 1999; Montoya-Burgos et al. 2003; Meunier and Duret 2004; Webster and Smith 2004; Spencer et al. 2006; Webster et al. 2006; Mancera et al. 2008; Escobar et al. 2011; Pessia et al. 2012; Lesecque et al. 2013; Williams et al. 2015; Halldorsson et al. 2016; Smeds et al. 2016; Keith et al. 2016; Long et al. 2018; Galtier et al. 2018; Smith et al. 2018). This meiotic distortion both mimics positive selection by increasing the fixation probability of G or C (i.e., S) over A or T (i.e., W) neutral alleles (Galtier and Duret 2007; Berglund et al. 2009; Ratnakumar et al. 2010), and promotes the fixation of slightly deleterious GC alleles (Duret and Galtier 2009; Glémin 2010; Necşulea et al. 2011; Lachance and Tishkoff 2014). A striking example of the latter effect is the mouse *Fxy* gene. This gene, which was recently (<1–3 Ma) translocated in the house mouse *Mus musculus* from the X-specific region to the highly recombining pseudoautosomal region (PAR), experienced a dramatic increase in W → S substitution rate in its part overlapping the PAR, at both coding and noncoding sites. This resulted in a >100-fold increase in amino acid substitution rate in the *M. musculus* lineage, illustrating how gBGC can promote the fixation of otherwise counter-selected W → S mutations (Perry and Ashworth 1999; Montoya-Burgos et al. 2003). Ratnakumar et al. (2010) showed that gBGC significantly affects the evolution of functional coding sequences in mammals, and can lead to patterns of evolution that can be mistaken for positive selection (Ratnakumar et al. 2010). Besides, it has been shown that gBGC can elevate the dN/dS ratio locally in specific genes in primates (Galtier et al. 2009; Berglund et al. 2009; Ratnakumar et al. 2010; Kostka et al. 2012) indicating that gBGC may not impact the evolution of selected versus neutral sites in the same way.

Somehow, the pattern reported in mammals does not seem to be observed in birds. An analysis of >8,000 genes in the *Ficedula* flycatcher lineage indicated that recombination and gBGC tend to decrease the dN/dS ratio (Bolívar et al. 2016). Another study focusing on passenger and band-tailed pigeons found a higher dN/dS ratio for substitutions opposed by gBGC and a lower one for substitutions promoted by gBGC (Murray et al. 2017), a result also confirmed by Bolívar et al. (2016). Besides, Corcoran et al. (2017) showed in great tits and zebra finches that ignoring the effect of gBGC can bias estimates of the DFE, *α* and *ω*_a_.

Interestingly, there are reasons to suspect that mammals and birds differ with respect to gBGC dynamics, due to a fundamental difference between these two taxa in the way recombination is controlled. In many mammals recombination hotspot location is determined by the *PRDM9* gene (Baudat et al. 2010; Myers et al. 2010; Parvanov et al. 2010; Sandor et al. 2012; Stevison et al. 2016; Baker et al. 2017). The *PRDM9* protein binds DNA through a highly variable tandem array of zinc fingers, and this participates to recruitment of the protein complex initiating recombination via double strand break. *PRDM9* evolves very rapidly (Oliver et al. 2009; Berg et al. 2011) and its binding motif experiences frequent changes (Brick et al. 2012), which result in a rapid turn-over in hotspot location, as demonstrated in primates and rodents (Lesecque et al. 2014; Baker et al. 2015; Latrille et al. 2017).

Birds, in contrast, lack the *PRDM9* gene (Baker et al. 2017). In this group, recombination hotspots seem to be mainly located upstream of genes. A recent study in flycatchers reports a correlation between hotspot location and CpG islands, CpG islands being themselves often located in promoter regions (Kawakami et al. 2017). Recombination rate is also linked to chromosome size (Consortium ICGS. 2004). The lack of *PRDM9* and the conserved karyotype of birds probably explain that the location of recombination hotspots is conserved across species (Mugal, Arndt, et al. 2013; Singhal et al. 2015). gBGC may thus have a particularly strong effect on bird genome evolution by persistently acting on specific genomic regions over long periods of time (Mugal, Arndt, et al. 2013). Moreover, phylogenetic analyses indicate that GC-content at putatively neutral sites is still increasing in avian genomes and has not yet reached its equilibrium (Webster et al. 2006; Nabholz et al. 2011; Weber et al. 2014). In contrast, GC-content at putatively neutral sites seems to be decreasing in primates (Duret et al. 2006). The distance between current GC-content and equilibrium GC-content has been shown to affect the estimation of the dN/dS ratio. Indeed, Bolívar et al. (2016) showed that the GC-content at 4-fold degenerated sites is further away from equilibrium than at 0-fold sites in flycatchers, leading to a stronger impact of gBGC on synonymous than on nonsynonymous substitutions, which entails a decrease of the dN/dS ratio (Bolívar et al. 2016).

The above reviewed literature suggests that the distinctive pattern of coding sequence evolution in mammals versus birds could be mediated by gBGC, and explained by the contrasting dynamics of recombination landscape between the two groups. This, however, is only a hypothesis requiring further corroboration. Firstly, the forces underlying this contrasted pattern are still difficult to understand theoretically (Galtier et al. 2009; Bolívar et al. 2016). Secondly, the dN/dS ratio has been measured and linked to gBGC using methods and gene sets that differ between studies, which can greatly influence the results. In particular, model choice and assumptions have been shown to potentially bias the estimation of dS, dN, and dN/dS (Guéguen and Duret 2017). Thirdly, we still lack a clear picture on how gBGC affects estimates of *α*, *ω*_a_ and *ω*_na_, besides an indication that not controlling for the effects of gBGC can lead to an overestimation of *α* (Corcoran et al. 2017). Finally, yet another level of complexity is added by the fact that recombination is expected to affect the synonymous and nonsynonymous substitution rate irrespective of gBGC. Recombination 1) could be mutagenic (Pratto et al. 2014; Arbeithuber et al. 2015), and 2) is expected to enhance the efficiency of natural selection by breaking linkage and Hill–Robertson interference (HRI, Hill and Robertson 1966).

Here, we investigated the influence of recombination and gBGC on adaptive and nonadaptive coding sequence evolution using two data sets composed of six species of catarrhine primates (mammals, with *PRDM9*) and six species of Galloanserae (birds, without *PRDM9*). We provide a detailed comparison of the influence of gBGC on coding sequence evolution by separately analyzing changes promoted by gBGC (W → S), changes countered by gBGC (S → W), and changes supposedly unaffected by gBGC, that is, GC-conservative ones (A ↔ T, C ↔ G). In both groups, we find that recombination strongly influences the synonymous substitution rate and the dN/dS ratio, particularly so for W → S changes, presumably reflecting the combined effect of gBGC and HRI. Contrary to what the current literature suggests, we report a roughly similar pattern in primates and Galloanserae, both showing a decrease of dN/dS with GC3. However, the shape of the relationship differs between the two taxa, likely reflecting differences in the dynamics of the recombination landscape. The analysis of GC-conservative synonymous substitutions reveals the existence of a mutagenic effect of recombination, which may also concern the other mutation types. Finally, we found that gBGC may lead to an overestimation of the adaptive substitution rate in both taxa.

## Results

### Primates and Galloanserae Alignments and SNP Calling

We used six species of primates (*Homo sapiens*, *Pan troglodytes*, *Papio anubis*, *Pongo abelii*, *Gorilla gorilla*, *Macaca mulatta*) and six species of Galloanserae (*Meleagris gallopavo*, *Phasianus colchicus*, *Pavo cristatus*, *Numida meleagris*, *Anas platyrhynchos*, *Anser cygnoides*) in which we could find either genomic or transcriptomic data in at least five individuals (Prado-Martinez et al. 2013; Teixeira et al. 2015; Wright et al. 2015; Xue et al. 2016). Orthogroups prediction yielded 8,604 orthogroups in primates and 4,439 orthogroups in Galloanserae. For each orthogroup, we estimated the branch specific dN, dS, and dN/dS ratio per category of mutation (W → S, S → W, GC-conservative) and the ancestral sequences at each internal node of the species tree. Synonymous and nonsynonymous Single Nucleotide Polymorphisms (SNPs) were called from polymorphism data and oriented using the predicted ancestral sequences, and classified as synonymous versus nonsynonymous, and W → S, S → W, or GC-conservative. The same genes were thus used for divergence and polymorphism analysis.

As a control for orientation errors we masked all sites in the alignment containing at least one CpG site. GC-conservative SNPs were far less numerous than W → S and S → W SNPs, with W → S and S → W SNPs representing on average 90% of the 6,447 (average per species) synonymous SNPs and 78% of the 2,750 (average per species) nonsynonymous SNPs.

### Correlation between Per-gene Recombination Rate and GC3

Using two available recombination maps (one for *H. sapiens* and one for *Gallus gallus*, see Materials and Methods) and the R package MareyMap, we estimated the per gene recombination rate (*r*) by comparing the genetic map with the physical position of genes. Mean *r* was quite different between the two species (*r* = 1.39 cM/MB with 95% confidence intervals of [0.52; 2.93] in *H. sapiens* and *r* = 3.98 cM/MB with 95% confidence intervals of [0.73; 12.43] in *G. gallus*). GC3 and *r* were significantly correlated in both species, with Spearman correlation coefficients of 0.39 (*P*-value < 2.2e–16) and 0.24 (*P*-value < 2.2e–16) for *G. gallus* and *H. sapiens* respectively (supplementary fig. S1, Supplementary Material online). We therefore used GC3 as a proxy of long-term recombination rate throughout the rest of the study.

Additionally, we estimated the correlation between GC3 and GC3*, the equilibrium GC-content at third codon position estimated under a model assuming nonstationary base composition. We found a significant positive correlation in Galloanserae (Spearman’s *R* = 0.38, *P*-value < 2.2e–16) and in primates but the correlation was weaker in the latter (Spearman’s *R* = 0.05, *P*-value = 4.6e–7). This is congruent with the suggestion that the recombination/gBGC landscape remains stable over long periods of time in birds (Mugal, Arndt, et al. 2013; Singhal et al. 2015), but evolves rapidly in primates (Lesecque et al. 2014).

### Influence of GC3 Level and Recombination Rate on Divergence Estimates

**Fig. 1.**
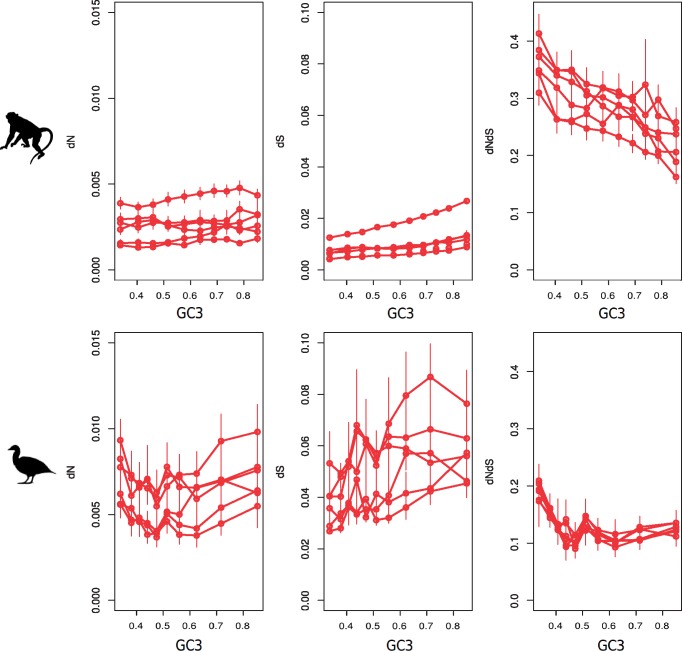
dN, dS, and dN/dS ratio against GC3 for each species for all substitutions taken together. Statistics are estimated under a model assuming base composition nonstationarity.

We binned orthogroups in ten sets of genes of equal size sorted by increasing GC3 or r and compared the lineage specific dN, dS and dN/dS ratio estimated assuming nonstationarity of base composition across bins (all changes). Figure 1 shows that in primates, the dN/dS ratio is negatively correlated with GC3 (this holds true when we use r instead of GC3, supplementary fig. S2, Supplementary Material online). This effect was significant in all primate species but *P. troglodytes* (see table 1). The relationship between dN/dS and GC3 was also negative in all six species of Galloanserae, but nonsignificant (table 1)*.* Besides, the shape of the decreasing relationship between dN/dS and GC3 was quite different between the two taxonomic groups. When we considered r instead of GC3 (supplementary table S3, Supplementary Material online), similar results were obtained, but significance was only reached in *P. abelii* after applying a False Discovery Rate (FDR) correction.

**Table 1.** Spearman Correlation Coefficients between GC3 and Divergence Estimates Obtained with a Model Assuming Nonstationarity.

Species	dN				dS				dN/dS			
	All	WS	SW	GC-conservative	All	WS	SW	GC-conservative	All	WS	SW	GC-conservative
*M. mulatta*	0.867**	0.915**	0.115	0.952**	1**	1**	1**	0.952**	–0.952**	–0.988**	–0.769*	–0.855**
*H. sapiens*	0.879**	0.139	0.818**	0.721*	0.988**	0.976**	0.964**	0.988**	–0.709*	–0.952**	0.188	–0.721*
*G. gorilla*	–0.103	–0.515	0.030	–0.067	0.939**	0.891**	0.539	0.903**	–1**	–0.976**	–0.539	–0.939**
*P. troglodytes*	0.927**	0.818**	0.939**	0.867**	1**	0.988**	0.891**	0.988**	–0.564	–0.939**	0.648*	–0.636
*P. anubis*	–0.467	–0.612	–0.636	0.091	0.867**	0.952**	0.661*	0.769*	–1**	–1**	–0.879**	–0.939**
*P. abelii*	0.830**	–0.356	0.830**	0.891**	1**	1**	1**	1**	–1**	–1**	–0.2	–0.939**
*M. gallopavo*	–0.369	–0.491	–0.042	–0.164	0.479	0.794**	–0.285	0.297	–0.564	–0.903**	0.309	–0.6
*N. meleagris*	–0.127	–0.806**	0.115	0.103	0.636	0.952**	–0.297	0.6	–0.430	–0.927**	0.236	–0.467
*P. cristatus*	–0.236	–0.648*	0.042	0.467	0.369	0.964**	0.552	0.769*	–0.273	–0.927**	–0.176	–0.624
*P. colchicus*	0.055	–0.212	0.321	0.079	0.745*	0.891**	0.345	0.455	–0.491	–0.806**	0.067	–0.297
*A. cygnoides*	0.588	0.624	0.467	0.673*	0.879**	0.988**	0.709	0.964**	–0.479	–0.939**	–0.151	–0.891**
*A. platyrhynchos*	0.527	0.479	0.503	0.612	0.891**	0.976**	0.333	0.927**	–0.527	–0.964**	0.055	–0.927**

Note.—Significance levels are showed with * before the FDR correction (False Discovery Rate), and with two shades of red (if the correlation is positive) or green (if the correlation in negative) after FDR correction (light: *P*-value < 0.05, dark: *P*-value < 0.01).

* *P*-value < 0.05.

** *P*-value < 0.01.

**Fig. 2.**
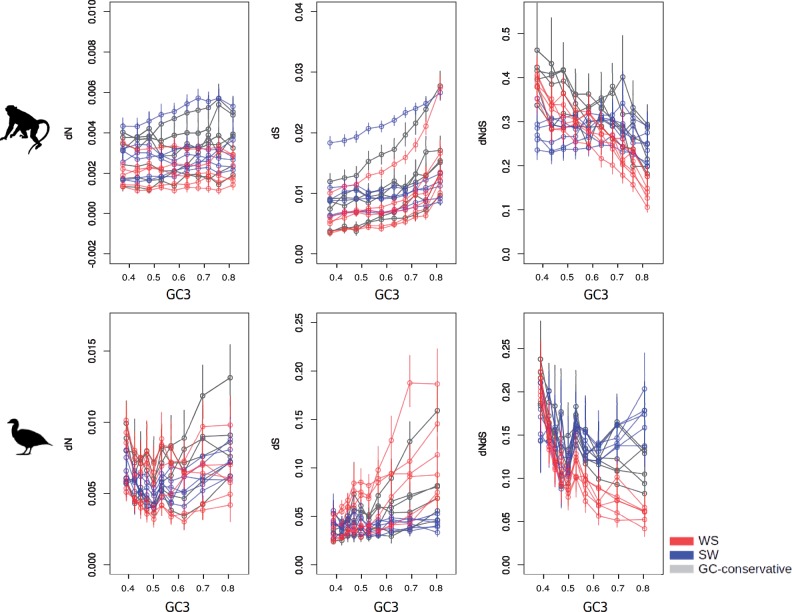
dN, dS, and dN/dS ratio against GC3 for each species and each type of substitutions (W → S, S → W, and GC-conservative substitutions). Statistics are estimated under a model assuming base composition nonstationarity.

To better understand these results, we separately analyzed W → S, S → W, and GC-conservative substitutions. We see in figure 2 that in both groups, the dN/dS ratio calculated from W → S substitutions (dN/dS_[W→S]_) decreases with GC3, an effect that is strong and significant in all twelve species (table 1)*.* This decrease in dN/dS_[W→S]_ with GC3 is due to a significant increase of dS_[W→S]_ for all species. dN_[W→S]_, in contrast, is only marginally influenced by GC3. When considering r instead of GC3, dS_[W→S]_ still strongly increases, but surprisingly, dN_[W→S]_ also increases with r, an effect that is significant for half of the species. dN/dS_[W→S]_ also decreases with r, and significantly so in eight species out of twelve (supplementary table S3, Supplementary Material online).

This suggests that gBGC has a strong influence on dN/dS_[W→S]_ by dramatically enhancing the fixation rate of W → S synonymous mutations, and much less that of W → S nonsynonymous mutations. HRI may be the mechanism that explains the decoupling between dS_[W→S]_ and dN_[W→S]_: as recombination increases, the increased efficiency of purifying selection presumably prevents slightly deleterious W → S mutations to come to fixation, thus counteracting the effect of gBGC on dN_[W→S]_. Alternatively, this could reflect a stronger effect of gBGC on synonymous sites compared with nonsynonymous sites because of a difference between the current GC-content and the equilibrium GC between sites—we further discuss this hypothesis below.

In contrast, no strong relationship was detected between dN/dS_[S→W]_ and GC3 or r. Interestingly, dS_[S→W]_ significantly increases with GC3 or r in primates (fig. 2, table 1). Under the hypothesis that both HRI and gBGC shapes the relationship between GC3 or r and divergence, we would expect both dN_[S→W]_ and dS_[S→W]_ to decrease with GC3 or r. To better understand the determinants of this surprising pattern, we analyzed the GC-conservative pattern of substitution. We found that dN/dS_[GC-conservative]_ globally decreases with GC3, significantly so in all primates but one, and in two Galloanserae. This is in agreement with the hypothesis that HRI affects the rate of fixation of slightly deleterious nonsynonymous mutations. Interestingly, we found that in most species dS_[GC-conservative]_, and to a lesser extent dN_[GC-conservative]_, are positively correlated with GC3 and r (table 1), which seems to imply the existence of a substantial mutagenic effect of recombination. This might explain why we do not observe a negative relationship between dN_[S→W]_ and dS_[S→W]_ and GC3.

We tested the robustness of these results to the codon model used in the estimation process of branch lengths and substitution parameters by reproducing the same divergence analysis with a model assuming stationarity of base composition. The results were very consistent between the two models (see supplementary figs. S3, S4 and tables S4 and S5, Supplementary Material online).

### Influence of GC3 Level and Recombination Rate on Polymorphism Estimates

**Fig. 3.**
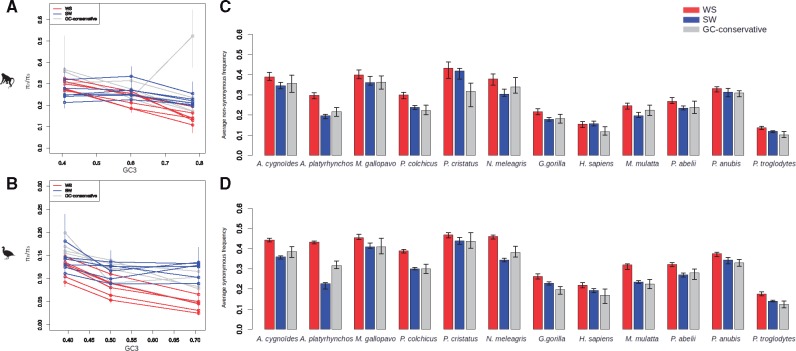
*π*_n_/*π*_s_ ratio against GC3 for each species (*A*: primates, *B*: Galloanserae) and each type of mutations (W → S, S → W, and GC-conservative mutations) and average nonsynonymous (*C*) and synonymous (*D*) allele frequency for each species for W → S, S → W, and GC-conservative SNPs (statistics estimated without masking CpG sites).

Within each species, SNPs were called from polymorphism data and classified as synonymous versus nonsynonymous, and W → S versus S → W versus GC-conservative (supplementary table S2, Supplementary Material online). We split the data set in three bins of genes of equal number of genes (fig. 3) and ten bins of equal number of SNPs (table 2) sorted according to GC3. We see in figure 3 and table 2 that *π*_n_/*π*_s[W→S]_ decreases with GC3, an effect which is significant in nine out of twelve species. This seems to be due to both a decrease of *π*_n[W→S]_ and an increase of *π*_s[W→S]_ with GC3 (table 2). S → W mutations and GC-conservative mutations show basically the same pattern, although less markedly than for W → S mutations. *π*_n_/*π*_s[GC-conservative]_ seems to decrease with GC3 as seen in figure 3, even if the correlation between *π*_n_/*π*_s[GC-conservative]_ and GC3 is significant for only four species. Figure 3*A* and *B* shows that the decay of *π*_n_/*π*_s_ with GC3 is steepest for W → S mutations, intermediate for GC-conservative mutations, and hardly significant for S → W mutations. These results are consistent with the divergence pattern, the GC-conservative pattern being intermediate between the W → S and S → W one. We found that in all twelve species W → S mutations segregate at a higher mean frequency than S → W and GC-conservative ones (fig. 3*C* and *D*), confirming the influence of gBGC (mean difference between W → S and S → W synonymous average SNP frequency is 0.069 [SD = 0.052], mean difference between W → S and GC-conservative synonymous average SNP frequency is 0.064 [SD = 0.026], mean difference between W → S and S → W nonsynonymous average SNP frequency is 0.041 [SD = 0.029], mean difference between W → S and GC-conservative nonsynonymous average SNP frequency is 0.047 [SD = 0028]).

**Table 2.** Spearman Correlation Coefficients between GC3 and *π*_n_, *π*_s_, *π*_n_/*π*_s_ Obtained without Masking CpG Sites.

Species	*π*_n_				*π*_s_				*π*_n_/*π*_s_			
	All	WS	SW	GC-conservative	All	WS	SW	GC-conservative	All	WS	SW	GC-conservative
*M. mulatta*	0.22	–0.34	0.34	0.23	0.90**	0.842**	0.6	0.70*	–0.77*	−0.78*	–0.22	–0.36
*H. sapiens*	0.61	0.50	0.69*	0.13	0.93**	0.38	0.83**	0.73*	–0.69*	–0.01	–0.45	–0.45
*G. gorilla*	0.18	0.12	0.12	0.18	–0.43	–0.57	0.28	–0.01	0.35	0.39	–0.054	0.2
*P. troglodytes*	–0.63	–0.79**	0.09	–0.39	0.91**	0.56	0.64*	–0.41	–0.96**	–0.86**	–0.66*	0.24
*P. anubis*	–0.5	–0.83**	0.06	0.2	0.93**	0.50	0.64*	0.57	–0.84**	–0.80**	–0.43	–0.29
*P. abelii*	0.63	0.15	0.68*	0.442	0.93**	0.47	0.93**	0.90**	–0.72*	–0.44	–0.44	–0.76*
*M. gallopavo*	–0.1	–0.78*	0.17	–0.55	0.83**	0.90**	0.24	–0.32	–0.75*	–0.93**	0.11	–0.33
*N. meleagris*	0.71*	–0.44	0.91**	0.47	0.96**	0.96**	0.92**	0.95**	–0.85**	–0.89**	–0.46	–0.61
*P. cristatus*	–0.12	–0.44	0.05	–0.45	0.86**	0.87**	0.90**	0.51	–0.88**	–0.83**	–0.55	–0.81**
*P. colchicus*	–0.7*	–0.90**	0.33	–0.76*	0.90**	0.63	0.73*	–0.56	–0.96**	–0.91**	–0.26	–0.56
*A. cygnoides*	0.34	–0.33	0.52	–0.22	0.91**	0.96**	0.90**	0.80**	–0.81**	–0.85**	–0.15	–0.78*
*A. platyrhynchos*	0.87**	–0.31	0.86**	0.52	0.89**	0.92**	0.89**	0.96**	–0.95**	–0.92**	–0.80**	–0.89**

Note.—Significance levels are showed with * before the FDR correction (False Discovery Rate), and with two shades of red (if the correlation is positive) or green (if the correlation in negative) after FDR correction (light: *P*-value < 0.05, dark: *P*-value < 0.01).

* *P*-value < 0.05.

** *P*-value < 0.01.

To minimize risks of orientation errors, we removed columns of the alignment containing at least one CpG site. Removing them (i.e., on average 6.3% of the sites in primates, and 5.9% in Galloanserae) drastically reduced *π*_s_ for each species (almost by a factor of two on average) and also led to the reduction of the significance level of the previous pattern for all mutation categories, but did not change our conclusions regarding *π*_n_/*π*_s_ (supplementary table S6, Supplementary Material online). However, the tendency for *π*_s[S→W]_ and *π*_s[W→S]_ to increase with GC3 (significantly for half of the species, both primates and birds), is far less present after masking CpG sites. SFS with and without CpG sites are shown in supplementary figures S8–S73, Supplementary Material online.

Splitting the data set in five bins instead of ten yielded qualitatively similar results (supplementary table S7, Supplementary Material online).

### Influence of GC3 Level on *α*, *ω*_a_, and *ω*_na_

**Fig. 4.**
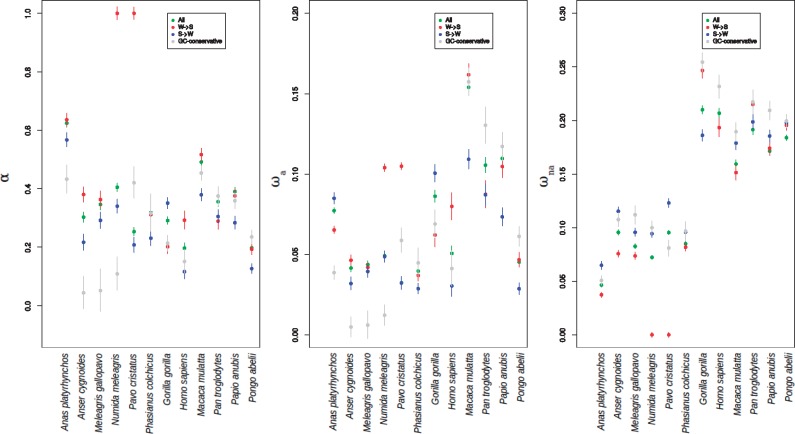
α, ωa and ωna estimates for each species and each type of mutations (all mutations, W→S, S→W and GC-conservative) using all genes. Statistics are obtained using the model “GammaZero”.

We estimated *α*, *ω*_a_ and *ω*_na_ on the whole sample of genes considering only GC-conservative mutations, S → W, W → S or all mutations at once using two different models for the DFE, namely GammaZero and GammaExpo (see Materials and Methods). Figure 4 shows that both *α*_[W→S]_ and *ω*_a[W→S]_ were higher than *α*_[S→W]_ and *ω*_a[S→W]_ in ten out of twelve species (binomial test *P*-value = 0.038; see also supplementary fig. S5, Supplementary Material online). *α*_[alll]_ and *ω*_a[all]_ were also higher than *α*_[GC-conservative]_ and *ω*_a[GC-conservative]_ in a majority of species, even if this was not significant (fig. 4 and supplementary fig. S5, Supplementary Material online). This indicates that gBGC could lead to an overestimation of the adaptive substitution rate. We checked that these results are robust to the number of individuals included in the analysis (supplementary fig. S6, Supplementary Material online).

Splitting the data set in ten bins of genes of equal number of SNPs sorted according to their GC3 level, we estimated the correlation between GC3 and *α*, *ω*_a_, and *ω*_na_. Our analysis did not allow to detect any significant effect of GC3 on the estimates of *ω*_a_ or *α* for any of the models (table 3, supplementary table S8, Supplementary Material online). For W → S and GC-conservative mutations, the correlation coefficient between *α* and GC3 was positive in a majority of species, but the relationship between *ω*_a_ and GC3 was not consistent across species. The fact that *ω*_a[GC-conservative]_ is positively correlated to GC3 in nine species out of twelve might indicate that an increased recombination rate leads to a greater efficiency of positive selection, but the effect is tenuous. The relation between *ω*_na_ and GC3 was found to be negative in all species when considering all mutations types together, and negative in ten out of twelve species for GC-conservative mutations, which might indicate that an increased recombination rate leads to a greater efficiency of purifying selection. However, this analysis is limited by a lack of statistical power due to the splitting of the data set in different bins of genes, resulting in a large sampling variance of estimates of *α*, *ω*_a_ and *ω*_na_. Splitting the data set in five bins yielded qualitatively similar results (supplementary table S9, Supplementary Material online).

**Table 3.** Spearman Correlation Coefficients between GC3 and α, ω_a_, and ω_na_ Estimates Obtained without Masking CpG Sites.

Species	*α*				*ω*_a_				*ω*_na_			
	All	WS	SW	GC-conservative	All	WS	SW	GC-conservative	All	WS	SW	GC-conservative
*M. mulatta*	0.41	0.24	0.0060	0.490	0.2	–0.24	–0.030	0.466	–0.27	–0.21	0.0424	–0.46
*H. sapiens*	0.28	0.32	0.35	0.28	0.33	0.24	0.357	0.381	–0.2	–0.34	–0.21	–0.38
*G. gorilla*	–0.06	0.12	0.52	0.042	–16	–0.090	0.49	0.17	–0.21	–0.054	–0.50	–0.17
*P. troglodytes*	0.38	0.393	0.381	0.35	0.6	0.187	0.49	0.35	–0.41	–0.33	–0.15	–0.35
*P. anubis*	0.09	0.0424	0.22	0.57	–0.08	0.22	0.187	0.57	–0.3	–0.15	–0.33	–0.57
*P. abelii*	0.26	0.22	0.32	–0.078	0.23	0.15	0.27	–0.29	–0.41	–0.2	–0.39	0.29
*M. gallopavo*	0.45	–0.20	–0.031	0.082	0.1	–0.15	0.0060	–0.28	–0.5	0.012	–0.11	–0.096
*N. meleagris*	0.03	0.143	–0.14	–0.13	–0.16	0.078	–0.30	–0.16	–0.13	0.056	0.14	0.093
*P. cristatus*	0.52	0.13	–0.032	0.30	0.62	–0.066	–0.22	0.22	–0.70*	–0.10	0.0064	–0.27
*P. colchicus*	0.04	0.20	–0.18	0.079	–0.39	–0.06	–0.16	0.030	–0.32	–0.27	0.018	–0.06
*A. cygnoides*	0.03	–0.055	–0.28	0.10	–0.18	0.006	–0.29	0.13	–0.19	–0.11	–0.10	–0.12
*A. platyrhynchos*	0.04	0.06	–0.03	0.57	–0.24	–0.53	–0.16	0.57	–0.56	–0.38	0.019	–0.57

Note.—*α*, *ω*_a_, and *ω*_na_ are obtained using the model GammaZero. Significance levels are showed with * before the FDR correction (False Discovery Rate), and with two shades of red (if the correlation is positive) or green (if the correlation in negative) after FDR correction (light: *P*-value < 0.05, dark: *P*-value < 0.01).

* *P*-value < 0.05.

** *P*-value < 0.01.

## Discussion

Here, we assessed the impact of recombination rate and gBGC on coding sequence evolution in two taxonomic groups, primates and Galloanserae, with contrasting recombination dynamics. We addressed this question by comparing estimates of dN/dS, *π*_n_/*π*_s_, *α*, *ω*_a_, and *ω*_na_ between bins of genes with different recombination rates and GC3, and by separately analyzing S → W, W → S, and GC-conservative changes.

### Recombination Influences Divergence and Polymorphism in a Roughly Similar Way in Birds and Primates

One of the most striking results we obtained is that all the measured variables, whether they are based on divergence data, polymorphism data, or both, were similarly influenced by recombination rate in the two taxonomic groups—despite some notable differences in the shape of the relationship between dN/dS and GC3 (see below). In particular, we showed for the first time that the dN/dS ratio in primates decreases with increasing GC3 and r, a result previously reported in passerines (Bolívar et al. 2016) and that we confirm here in Galloanserae, despite the lack of significance of the signal. Previous studies in primates have indicated that gBGC promotes a local increase in the dN/dS ratio and can mislead the inference of positive selection (Galtier et al. 2009; Berglund et al. 2009; Ratnakumar et al. 2010; Kostka et al. 2012). Those studies focused on a small subset of genes a priori identified on the basis of their high dN/dS. Our results indicate that the positive effect of gBGC on dN/dS is only local/transient and that, in contrast, the global pattern is a negative relationship between recombination rate and dN/dS in primates. A significant negative correlation between dN/dS and the equilibrium GC-content has also been observed in a data set of 17 nuclear protein-coding genes in 73 placental taxa (Lartillot 2013), consistent with our results. As for birds, our results are consistent with previous analyses in flycatchers (Bolívar et al. 2016).

That said, our analysis revealed some differences between the two taxonomic groups. Most importantly, the shape of the relationship between dN and dN/dS and GC3 differs between primates and Galloanserae (fig. 1). We observe that in Galloanserae, dN varies nonmonotonically with GC3. dN decreases with GC3 at low GC3 values, but increases with GC3 at high GC3 values. dN/dS also shows a sharp decline at low GC3 values and then plateaus. The initial decrease of dN and dN/dS seems to depict the effect of HRI: in very low recombining regions, selection against deleterious mutations is poorly efficient. When recombination increases, selection becomes more efficient, until a recombination rate threshold is reached, above which interferences become negligible (Kliman and Hey 1993; Comeron and Kreitman 2002; Corbett-Detig et al. 2015). The increase in dN with GC3 above this threshold can be interpreted as reflecting the mutagenic effect of recombination, as it affects both dN_[W→S]_ and dN_[GC-conservative]_ and is not perceptible in the dN/dS analysis (fig. 2).

Interestingly, the six species of primates show a different pattern, that is, a gradual decrease of dN/dS with GC3. We suggest that this might be explained by the variation in time of recombination map in primates due to the presence of *PRDM9*. Let us assume, as discussed above, that HRI affects dN when the recombination rate is below some threshold (low-r state), but negligibly so when the recombination rate is above this threshold (high-r state). If r varies in time, then the time-averaged HRI effect for a given gene is expected to reflect the proportion of time spent by this gene in the low-r state as species were diverging. Said differently, we suggest that GC3 and dN/dS are expected to recapitulate the long-term effect of gBGC and HRI, respectively, which in primates vary continuously across genes due to the temporal dynamics of recombination rate.

Additionally, some species of primates show an increase of dN_[S→W]_ and dS_[S→W]_ with GC3 and r, whereas this is not observed in birds (table 1 and fig. 2). This could reflect an effect of back S → W mutations after gBGC has been turned off due to the shifting of recombination hotspot location in primates—a process that might be less prevalent in birds due to the stability of the recombination landscape. This is an interesting hypothesis that would deserve to be investigated further.

Bolívar et al. (2016), finally, concluded that the impact of gBGC on the dN/dS ratio may be mainly governed by the difference between the current GC-content and GC*. They suggest that gBGC leads to a reduced dN/dS in high recombining regions in the flycatcher lineage due to current GC-content being lower than GC*, and more so at synonymous than nonsynonymous sites. Here, we found a decreasing GC3 in primates (GC3*–GC3∼ –0.14 [–0.5; 0.25] on average) and an increasing one in Galloanserae (GC3*–GC3∼ 0.05 [–0.31; 0.28] on average) towards equilibrium, a result consistent with previous studies (Duret et al. 2006; Nabholz et al. 2011; Weber et al. 2014). These differences may contribute to the observed differences of behavior of the dN/dS ratio between primates and Galloanserae.

### Selection versus gBGC: Who Wins?

Here, we show that there is a stronger influence of gBGC on synonymous sites than on nonsynonymous sites. To explain this result, we suggest that another evolutionary force may compensate for the effects of gBGC on nonsynonymous sites. One good candidate here is HRI, as suggested by our results concerning GC-conservative changes. Indeed, *π*_n_/*π*_s[GC-conservative]_, as well as dN/dS_[GC-conservative]_ and the nonadaptive substitution rate *ω*_na[GC-conservative]_, decrease with GC3 in most species—although not always significantly. This is consistent with the HRI hypothesis, and with previous studies empirically demonstrating a link between recombination rate and genetic diversity in primates and birds (Spencer et al. 2006; Mugal, Nabholz, et al. 2013; Corbett-Detig et al. 2015). The intensity of the decrease of *π*_n_/*π*_s_ and *ω*_na_ with r is not the same for all mutations types, though, suggesting that gBGC also plays a role here. Anyway, this result confirms that in both taxonomic groups purifying selection is more efficient in highly recombining regions of the genome.

Alternatively, Bolívar et al. (2016) suggested that the influence of gBGC on neutrally and selected sites mainly depends on current synonymous and nonsynonymous GC-content, and the distance to their respective equilibrium (GC*). They found that in flycatchers the relationship between dN/dS and recombination rate mainly reflect the greater distance between current and equilibrium GC-content at 4-fold than at 0-fold degenerated sites (Bolívar et al. 2016). Using GC2 as a proxy for the nonsynonymous GC-content, we found that in the two taxonomic groups current nonsynonymous GC-content is far away from its equilibrium. In particular, in Galloanserae, we estimated a greater distance between GC and GC* at nonsynonymous sites than at synonymous sites, suggesting that Bolívar’s explanation does not apply here.

gBGC has been termed the “Achilles’ heel” of the genome. It has been shown that an elevated recombination rate could locally decrease the efficiency of purifying selection due to the fixation of W → S deleterious mutations through gBGC (Dreszer et al. 2007; Duret and Galtier 2009). Glémin (2010) developed a population genetics model including gBGC and showed that the interaction between gBGC and selection has important consequences on load and inbreeding depression. Here, we show that this effect, which can be locally strong in the vicinity of recently active recombination hotspots, does not dominate when one considers all genes and a longer time scale. We suggest that recombination influences synonymous and nonsynonymous substitution rates via a combination of the effects of gBGC and HRI. The former is demonstrated by the distinctive patterns we report between W → S and S → W changes, and the latter by the existence of an effect of GC3 or r on dN/dS_[GC-conservative]_.

Interestingly, we observe that the dN/dS ratio varies much between bins of genes and categories of changes—up to a factor of four (fig. 2). These differences are presumably independent of the selective constraints acting on the corresponding genes. This implies that controlling for recombination rate is of utmost importance when using dN/dS as a proxy for the extent of selective pressure acting on a gene.

### A Mutagenic Effect of Recombination?

We report a positive correlation between dS_[GC-conservative]_ and both r and GC3, which reveals the existence of a mutagenic effect of recombination. A similar result was reported in flycatcher (Bolívar et al. 2016), but little discussed. In humans, several studies have previously reported such a phenomenon (Pratto et al. 2014; Arbeithuber et al. 2015; Smith et al. 2018). Pratto et al. (2014) specifically examined the mutation process around recombination hotspots by analyzing rare variants. They found that G ↔ A, C ↔ T, and G ↔ C mutations were enriched around recombination hotspots, G → A and C → T being the most frequent (Pratto et al. 2014). Arbeithuber et al. (2015) reported from sperm typing analysis that mutations appearing simultaneously with cross-over events are enriched in S → W changes. More recently, Smith et al. (2018) analyzed father/mother/child trios and showed that both the S → W and the GC-conservative mutation rate are positively correlated with recombination rate, whereas results were less consistent across data set as far as the W → S rate was concerned.

In view of these recent results, it is quite plausible that the effect we detect on GC-conservative mutations is driven by a G ↔ C and A ↔ T mutagenic effect of recombination. Additionally, the fact that we do not detect a negative relationship between GC3 and dS_[S→W]_ or dN_[S→W]_ may be due to the fact that S → W mutations are submitted to an enhanced recombination-linked mutagenic effect as reported in Pratto et al. (2014) and Arbeithuber et al. (2015), which counterbalances the effect of gBGC. Finally, we cannot exclude the existence of an enhanced mutation associated with recombination regarding W → S mutations, as Pratto et al. (2014) detected such an effect. However, it seems weaker than the S → W mutation bias in view of the results of Arbeithuber et al. (2015) and Smith et al. (2018).

### Influence of gBGC on the Adaptive Substitution Rate

The comparison of *ω*_a_ and *α*, computed from the total set of genes for different mutation categories reveals that estimates of the adaptive substitution rate are lower for S → W mutations than for W → S ones, in agreement with the suggestion that gBGC mimics positive selection in increasing the fixation probability of neutral and slightly deleterious W → S mutations. Additionally, we found that *ω*_a_ and *α* were often higher when considering all mutations than when considering only GC-conservative ones, confirming that gBGC tends to entail an increase of the estimated adaptive substitution rate. This is in line with what was observed in great tits and zebra finch, where using only CG-conservative mutations led to a decreased estimate of *α* (Corcoran et al. 2017). Our analysis, however, did not allow to accurately validate the prediction that HRI leads to positive selection being less efficient in low recombining regions compared with highly recombining regions (whereas this was shown in a fungal pathogen, Grandaubert et al. 2018, and in *Drosophila*, Castellano et al. 2016)—probably due to a lack of power.

Our results regarding the influence of recombination and gBGC on *ω*_a_ and *α* may be explained by the fact that the estimation process is sensitive to some technical biases, in particular orientations errors (in this case, low frequency W → S SNPs misattributed as high frequency S → W SNPs and conversely). As some of the SFS in Galloanserae showed an unusual shape (*M. gallopavo, P. cristatus*, *N. meleagris, A. cygnoides*, supplementary figs. S8–S73, Supplementary Material online), we took particular care to remove any sources of such errors. We incorporated the so-called r_i_’s nuisance parameters (Eyre-Walker et al. 2006; Eyre-Walker and Keightley 2009), to be optimized along side with DFE parameters in the DFE-*α* method (see Materials and Methods). These parameters are intended to capture a wide range of effects that would distort the shape of SFS, including orientation errors, as long as they influence similarly the synonymous and nonsynonymous SFS. We also removed CpG sites from the sequences, as CpG hypermutability may make unfolded SFS very sensible to polarization errors. This did not significantly affect the results (supplementary figs. S8–S73, Supplementary Material online), suggesting that the pattern we are reporting regarding *ω*_a_ and *α* reflects a real effect of gBGC on coding sequence evolution.

## Conclusions

Our analysis revealed a substantial effect of recombination and gBGC on the rate of coding sequence evolution in primates and Galloanserae, with dN/dS varying by up to 4-fold between categories of genes and base changes irrespective of gene function. We report an increase in dS and a decrease in dN/dS with GC3 and recombination rate, which are particularly strong as far as W → S mutations are concerned, demonstrating a combined influence of gBGC and HRI. This pattern is reported in mammals as well as in birds, despite some differences in the dynamic of the influence of GC3 or r. This suggests that the presence/absence of *PRDM9* is not as strong a predictor of the long-term evolutionary pattern of coding sequences as we hypothesized, but that it may still lead to differences in the dynamic of the impact of recombination on coding sequences between primates and birds. Overall, our analysis demonstrates a complex effect of recombination on molecular evolution, which should be appropriately taken into account when interpreting patterns of coding sequence variation among genes and genomes.

## Materials and Methods

### Sequence Data

We used six species of primates and six species of Galloanserae for which we had >5 individuals: *H. sapiens* (19 individuals), *P. troglodytes* (20 individuals), *P. anubis* (5 individuals), *P. abelii* (10 individuals), *G. gorilla* (20 individuals), *M. mulatta* (19 individuals), and *M. gallopavo* (10 individuals), *P. colchicus* (10 individuals), *P. cristatus* (10 individuals), *N. meleagris* (10 individuals), *A. platyrhynchos* (10 individuals), *A. cygnoides* (10 individuals).

For primates reference genomes, assemblies and annotations files were downloaded from Ensembl (release 89). We kept only “*CDS*” reports in the annotations files, corresponding to coding exons, which were annotated with the automatic *ensembl* annotation pipeline, and the havana team for *H. sapiens*. Raw genomic reads for each primate individuals were retrieved from various Bioproject of SRA (see supplementary table S1, Supplementary Material online). We used trimmomatic to remove Illumina adapters and trimmed low-quality reads (i.e., with an average base quality below 20), and kept only reads longer than 50 bp.

For Galloanserae, we retrieved RNA-seq reads from SRA Bioproject PRJNA271731, generated in a previous study (Wright et al. 2015). We used trimmomatic to remove Illumina adapters as well as reads with a quality below 30. We constructed de novo transcriptome assemblies for each species following strategies B in (Cahais et al. 2012), using Abyss (Simpson et al. 2009) and Cap3 (Huang and Madan 1999). Open reading frames (ORFs) were predicted using the Trinity package (Grabherr et al. 2011). Contigs carrying ORF shorter than 150 bp were discarded.

### SNP Calling

Primates reads were mapped using Burrow Wheeler Aligner (BWA) software (Li and Durbin 2009) on the complete reference assembly (version 0.7.12-r1039). We filtered out hits with mapping quality below 20 and removed duplicates, and we extracted mapping hits corresponding to regions containing coding sequences according to the annotated reference assembly. This was done to avoid calling SNPs on the whole genome, which is heavily time consuming and useless in the present context. We called SNPs using a pipeline based on GATK (v3.8-0-ge9d80683). Roughly, this pipeline comprised two rounds of variant calling separated by a base quality score recalibration. Variant calling was first run on every individuals from every species using HaplotypeCaller (–emitRefConfidence GVCF –genotyping_mode DISCOVERY -hets 0.001). The variant callings from all individuals of a given species were then used to produce a joint genotype using GenotypeGVCFs. Indels in the resulting vcf files were then filtered out using vcftools. The distributions of various parameters associated with SNPs were then used to set several hard thresholds (i.e., Quality by Depth < 3.0; Fisher Strand > 10; Strand Odds Ratio > 3.0; MQRootMeanSquare < 50; MQRankSum < –0.5; ReadPosRankSum < –2.0) in order to detect putative SNP-calling errors using VariantFiltration. This erroneous SNPs were then used for base quality score recalibration of the previously created mapping files using BaseRecalibrator. These mappings with recalibrated quality scores were then used to recall variants (HaplotypeCaller), to reproduce a joint genotype (GenotypeGVCFs, –allsites) and to reset empirical hard thresholds (i.e., same values as above, except for Quality by Depth < 5.0). The obtained vcf files were converted to fasta files using custom python scripts while discarding exons found on both mitochondrial and sexual chromosomes and while filtering out additional SNPs. We removed SNPs with a too high coverage (thresholds were empirically set for each species), with a too low coverage (i.e., 10× per individual) and with a too low genotype quality per individual (i.e., <30).

For Galloanserae, filtered RNA-seq reads were mapped to predicted cDNAs with BWA (Li and Durbin 2009). Contigs with a per individual average coverage below ×2.5 were discarded. Diploid genotypes were called according to the method described in Tsagkogeorga et al. (2012) (model M1) via a the software reads2snps. This software calls a genotype at each site with a minimum of 10 reads and calculates the posterior probability of each possible genotype in the maximum likelihood framework. Genotypes supported by a posterior probability >95% are retained, otherwise missing data are called. We used a version of the method which accounts for between-individual, within-species contamination as introduced in Ballenghien et al. (2017), using the -contam = 0.1 option, which means assuming that up to 10% of the reads assigned to one specific sample may actually come from a distinct sample, and only validating genotypes robust to this source of uncertainty.

### Orthology Prediction

For primates, we extracted the 1-to-1 orthologous prediction of the six species from the OrthoMaM database (Ranwez et al. 2007; Douzery et al. 2014).

For Galloanserae, we translated the obtained CDS into proteins and predicted orthology via OrthoFinder (Emms and Kelly 2015) that uses a proteic BLAST (Basic Local Alignment Search Tool). For this specific step, we added coding sequences of the chicken *G. gallus*, extracted from the reference genome inEnsembl (release 89). Indeed, RNA-seq assemblies are very fragmented, and chicken CDS were on average longer than contigs assembled via the de novo transcriptome assemblies. Including long CDS allowed OrthoFinder to group several RNA-seq contigs of a same species into one orthogroup, allowing us to concatenate such contigs into one longer sequence after checking that they were not overlapping and thus improving the orthologous detection. We kept only orthogroups that included all species.

For both groups, we aligned the orthologous sequences via MACSE (Multiple Alignment for Coding Sequences, Ranwez et al. 2011).

### Per Gene Recombination Rate Computation

We used the R package MareyMap (R version 3.4.3 [30/11/2017]) to perform a Loess interpolation method (LOcally WEighted Scatterplot Smoothing; Rezvoy et al. 2007) with a span of 0.2 on two recombination maps (one for *H. sapiens* and one for *G. gallus*) to estimate the per gene recombination rate (r) by comparing the genetic map with the physical position of genes. We used the recombination map of *H. sapiens* from the Phase 2 HapMap project (HapMap release 22, NCBI 36) that comprises over 3.1 millions SNPs (*Frazer et al. 2007*), and the recombination map of *G. gallus* that comprises 9.268 SNPs (Groenen et al. 2008).

### Divergence and Polymorphism Statistics Computation

For both taxonomic groups, we used the bppml program and a modified version of mapNH (http://biopp.univ-montp2.fr/wiki/index.php/Main_Page, last accessed January 23, 2019; Romiguier et al. 2012; Guéguen et al. 2013) to estimate the synonymous and nonsynonymous substitution rate (dS, the number of synonymous substitutions per synonymous site and dN, the number of nonsynonymous substitutions per nonsynonymous site) per branch by substitution mapping under the Nielsen–Yang model (Nielsen and Yang 1998). We used the tree topologies presented in supplementary figure S7, Supplementary Material online.

We tested and compared both a model assuming a stationary base composition and a model assuming a nonstationary base composition. This was motivated by the results of Guéguen and Duret (2017) stating that estimates of dN, dS and dN/dS can be biased when using standard methods assuming sequence stationarity, this bias being influenced by the evolution of GC3 in particular (Guéguen and Duret 2017). We did so for three categories of substitution: W → S, S → W, and GC-conservative.

For each sequence we estimated the nonsynonymous and synonymous number of sites for each of those categories to normalize substitutions counts, using an in-house script that counts up mutational opportunities of each mentioned category of mutation under a neutral model assuming a transition–transversion ratio. The principle of this count is as follow: for each site, there are three possible alternative states which are examined. We estimate the probability to mutate to either of the three possible states using a ratio of transition over transversion parameter (estimated from the data). We then we add up those probabilities across sites, separating possible changes that are synonymous from possible changes that are nonsynonymous along the gene. When counting only W → S (or S → W or GC-conservative) sites, we use the same strategy but restrict the counts to the relevant alternative states.

We then computed dN, dS, and dN/dS estimates for bins of genes defined according to GC3 level or per-gene recombination rate (ten bins of equal size) by summing substitutions and number of sites across genes and then dividing the sum of substitutions by the sum of number of sites. Ninety-five percent confidence intervals were determined by bootstrapping genes (1,000 replicates).

For each alignment, we estimated ancestral sequences at each node of the tree with the Bio ++-based SeqAncestor program (Guéguen et al. 2013). Ancestral sequences were then used to orientate mutations, so that we could then compute nonsynonymous (*π*_n_) and synonymous (*π*_s_) nucleotide diversity and SFS for three bins of genes of equal size and ten bins of genes of equal number of SNPs, and each mutation category, via an in-house script. Ninety-five percent confidence intervals on *π*_n_ and *π*_s_ were determined by bootstrapping genes (100 replicates). We applied the same protocol after removing columns of the alignments that contains at least one CpG site.

### Adaptive and Nonadaptive Substitution Rate Computation

We estimated *α*, *ω*_a_, and *ω*_na_ using the method of (Eyre-Walker and Keightley 2009) as implemented in Galtier (2016). It models the DFE of deleterious nonsynonymous mutations as a negative Gamma distribution, which is fitted to the synonymous and nonsynonymous SFS computed for a set of genes.

We accounted for recent effects (demographic or other) that could distort the SFS relative to the neutral expectation in an equilibrium Wright–Fisher population by adjusting the so-called r_i_’s nuisance parameters alongside with the DFE parameters. They are adjusted for each allele frequency class in the SFS, and multiply both the synonymous and the nonsynonymous expected number of SNP (Eyre-Walker et al. 2006).

We computed *α*, *ω*_a_, and *ω*_na_ for each terminal branches of the tree for primates and Galloanserae, for ten bins of genes of equal number of SNPs sorted depending on their GC3, and each category of mutation and substitution (W → S, S → W, and GC-conservative). We tested two different models to fit the DFE that are described in Galtier (2016). Briefly, the GammaZero models a negative DFE only, as a Gamma distribution. The GammaExpo model contains a negative Gamma DFE as well as a positive DFE modeled as an exponential. Confidence intervals correspond to the maximum likelihood confidence intervals computed during the optimization step using the Newton–Raphson method (defined as values of *α* and *ω*_a_ for which the log-likelihood was within two units of its maximum).

## Supplementary Material

Supplementary data are available at *Molecular Biology and Evolution* online.

## Supplementary Material

Supplementary Data
