# Supplementary material for: Influence of Recombination and GC-biased Gene Conversion on the Adaptive and Nonadaptive Substitution Rate in Mammals versus Birds
Source: Mol Biol Evol. 2018 Dec 27;36(3):458–71. doi: 10.1093/molbev/msy243 (PMC6389324; doi:10.1093/molbev/msy243)
Supplement: Supplementary Data [file msy243_supp.zip › Supplementary_Material.pdf]

Supplementaty Material

Species	Bioproject	data_type	number_of_individuals	publication
Gorilla gorilla	PRJNA189439	Genome	20	Prado-Martinez et al. 2013
Homo sapiens	PRJEB8350	Exome	19	Teixeira et al. 2015
Pan troglodytes	PRJEB8350	Exome	20	Teixeira et al. 2015
Papio anubis	PRJNA54005	Genome	5	unpublished baboon genome project
Pongo abelii	PRJNA189439 and PRJEB1675	Genome	10	Prado-Martinez et al. 2013 and -
Macaca mulatta	PRJNA251548	Exome	20	Xue et al. 2016
Meleagris gallopavo	PRJNA271731	RNA_seq	10	Wright et al. 2015
Phasianus colchicus	PRJNA271731	RNA_seq	10	Wright et al. 2015
Pavo cristatus	PRJNA271731	RNA_seq	10	Wright et al. 2015
Numida meleagris	PRJNA271731	RNA_seq	7	Wright et al. 2015
Anas platyrhynchos	PRJNA271731	RNA_seq	10	Wright et al. 2015
Anser cygmoides	PRJNA271731	RNA_seq	10	Wright et al. 2015

Table S1: Details of the Bioprojects used in this study to retrieve reads data.

species	dN				dS				dNdS			
	all	WS	SW	GC-conservative	all	WS	SW	GC-conservative	all	WS	SW	GC-conservative
M. mulatta	0.867**	0.794**	0.782*	0.769*	0.83**	0.830**	0.939**	0.806**	-0.685*	-0.782*	-0.479	-0.588
H. sapiens	0.806**	0.855**	0.515	0.806**	0.988**	0.867**	0.927**	0.915**	-0.685*	-0.467	-0.321	-0.818**
G. gorilla	0.224	0.539	-0.127	0.248	0.818**	0.769*	0.624	0.782*	-0.624	-0.758*	-0.515	-0.576
P. troglodytes	0.782*	0.648*	0.842**	0.696*	0.976**	0.988**	0.952**	0.842**	-0.430	-0.842**	0.224	-0.176
P. anubis	0.636**	0.758*	0.406	0.563	0.818**	0.830**	0.782*	0.830**	-0.721*	-0.515	-0.418	-0.672*
P. abelii	0.624	0.358	0.685*	0.624	0.830**	0.806**	0.806**	0.685*	-0.927**	-0.879**	-0.467	-0.624
M. gallopavo	0.273	0.164	-0.127	0.176	0.455	0.794**	-0.552	0.745*	-0.006	-0.733*	0.418	-0.345
N. meleagris	0.685*	0.830**	0.272	0.006	0.915**	0.988**	-0.2	0.709*	-0.612	-0.879**	0.358	-0.6
P. cristatus	0.818**	0.758*	0.394	0.733*	0.818**	0.842**	-0.261	0.624	-0.261	-0.818**	0.442	-0.091
P. colchicus	0.552	0.176	0.709*	0.515	0.733*	0.830**	0.418	0.673*	-0.503	-0.636*	-0.151	-0.358
A. cygnoides	0.745*	0.830**	0.564	0.696*	0.891**	0.915**	-0.285	0.6	0.176	-0.358	0.430	0.054
A. platyrhynchos	0.564	0.164	0.745*	0.576	0.757*	0.891**	-0.115	0.867**	-0.091	-0.794**	0.539	-0.733*

Table S3: Spearman correlation coefficients between r and divergence estimates obtained with a model assuming non-stationarity.

Significance levels are showed with * (* p-value< 0.05, ** p-value < 0.01) before the FDR correction, and with two shades of red (if the correlation is positive) or green (if the correlation in negative) after FDR correction (light :p-value< 0.05, dark : p-value< 0.01).

Supplementaty Material

species	dN				dS				dNdS			
	all	WS	SW	GC-conservative	all	WS	SW	GC-conservative	all	WS	SW	GC-conservative
M. mulatta	0.903**	0.952**	-0.139	0.927**	0.939**	0.988**	0.055	0.939**	-0.855**	-0.988**	-0.418	-0.964**
H. sapiens	0.879**	0.491	0.842**	0.915**	1**	0.988**	0.758*	1**	-0.588	-0.988**	0.588	-0.636
G. gorilla	-0.442	-0.067	-0.782	0.188	0.612	0.927**	-0.6	0.418	-0.976**	-0.989**	-0.939**	-0.915**
P. troglodytes	0.915**	0.673*	0.939**	0.891**	1**	1**	0.867**	1**	#VALEUR !	-0.976**	0.782*	-0.552
P. anubis	-0.503	-0.321	-0.915**	0.697*	0.939**	1**	-0.891**	0.939**	-0.976**	-0.951**	-0.564	-0.988**
P. abelii	0.891**	0.406	0.769*	0.939**	1**	1**	0.988**	0.988*	-0.976**	-0.988**	0.673*	-0.952**
M. gallopavo	-0.467	-0.588	-0.188	-0.673*	0.309	0.915**	-0.745*	-0.588	-0.709*	-0.903**	0.3812	0.479
N. meleagris	-0.285	-0.6	0.042	-0.539	0.685*	0.964**	-0.903**	-0.563	-0.430	-0.915**	0.624	0.358
P. cristatus	-0.248	-0.394	0.236	-0.491	0.394	0.976**	-0.079	-0.503	-0.551	-0.952**	0.285	0.418
P. colchicus	-0.067	-0.030	0.2	-0.418	0.806**	0.976**	0.212	-0.503	-0.636	-0.939**	0.091	0.527
A. cygnoides	0.576	0.745*	0.273	-0.139	0.867**	1**	-0.127	-0.067	-0.6	-0.915**	0.442	0.176
A. platyrhynchos	0.527	0.648*	0.345	-0.091	0.891**	0.988**	-0.624	0.054	-0.6	-0.951**	0.564	-0.212

Table S4: Spearman correlation coefficients between GC3 and divergence estimates obtained with a model assuming stationarity.

Significance levels are showed with * (* p-value< 0.05, ** p-value < 0.01) before the FDR correction, and with two shades of red (if the correlation is positive) or green (if the correlation in negative) after FDR correction (light :p-value< 0.05, dark : p-value< 0.01).

species	dN				dS				dNdS			
	all	WS	SW	GC-conservative	all	WS	SW	GC-conservative	all	WS	SW	GC-conservative
M. mulatta	0.685*	0.673*	0.479	0.697*	0.794**	0.782*	0.673*	0.830**	-0.855**	-0.964**	-0.564	-0.636
H. sapiens	0.564	0.636	0.418	0.539	0.855**	0.758*	0.842**	0.709*	-0.806**	-0.745*	-0.285	-0.769*
G. gorilla	0.709*	0.818**	0.115	0.806**	0.927**	0.903**	0.733*	0.915**	-0.867**	-0.660*	-0.842**	-0.758*
P. troglodytes	0.721*	0.721*	0.745*	0.672*	0.952**	1**	0.964**	0.879**	-0.212	-0.879**	0.236	-0.564
P. anubis	0.261	0.442	0.321	0.455	0.758*	0.879**	0.661*	0.673*	-0.769*	-0.830**	-0.479	-0.794**
P. abelii	0.6	0.624	0.685*	0.806**	0.842**	0.891**	0.697*	0.842**	-0.927**	-0.927**	-0.018	-0.769*
M. gallopavo	-0.164	-0.079	-0.333	-0.564	0.503	0.782*	-0.721*	-0.782*	-0.612	-0.782*	0.321	0.152
N. meleagris	-0.382	0.369	-0.467	-0.769*	0.806**	0.988**	-0.673*	-0.685*	-0.612	-0.903**	0.176	-0.018
P. cristatus	0.491	0.733*	0.067	0.055	0.758*	0.915**	-0.394	-0.491	-0.297	-0.867**	0.564	0.721*
P. colchicus	-0.018	-0.006	0.381	-0.418	0.830**	0.891**	0.224	-0.685*	-0.552	-0.636	-0.164	0.103
A. cygnoides	0.612	0.745*	0.285	-0.127	0.867**	0.952**	-0.842**	-0.564	0.091	-0.552	0.661*	0.224
A. platyrhynchos	0.176	0.394	0.709*	-0.321	0.636	0.903**	-0.2	0.685*	-0.636	-0.855**	0.6	-0.769*

Table S5: Spearman correlation coefficients between r and divergence estimates obtained with a model assuming stationarity.

Significance levels are showed with * (* p-value< 0.05, ** p-value < 0.01) before the FDR correction, and with two shades of red (if the correlation is positive) or green (if the correlation in negative) after FDR correction (light :p-value< 0.05, dark : p-value< 0.01).

Supplementaty Material

species	π_n			π_s			π_n/π_s		
	WS	SW	GC-conservative	WS	SW	GC-conservative	WS	SW	GC-conservative
M. mulatta	-0.939**	-0.454	-0.527	-0.236	0.406	0.236	-0.915**	-0.333	-0.442
H. sapiens	-0.3	-0.030	-0.483	0.483	0.139	0.3	-0.216	0.103	-0.7*
G. gorilla	-0.757*	-0.6	-0.595	-0.612	-0.612	0.071	-0.115	-0.830**	-0.833*
P. troglodytes	-0.951**	-0.745*	-0.683	-0.648*	-0.575	-0.383	-0.890**	-0.733*	-0.283
P. anubis	-0.890**	-0.563	-0.818**	-0.418	0.163	0.333	-0.684*	-0.660*	-0.527
P. abelii	-0.612	-0.272	-0.103	-0.296	0.151	-0.151	-0.248	-0.806**	-0.212
M. gallopavo	-0.854**	0.151	-0.478	-0.333	0.224	-0.2	-0.696*	0.139	-0.284
N. meleagris	-0.672*	0.866**	-0.321	0.709*	0.963**	0.381	-0.842**	-0.430	-0.624
P. cristatus	-0.793**	-0.527	-0.454	0.684*	0.745*	0.490	-0.903**	-0.539	-0.539
P. colchicus	-0.890**	0.418	-0.806**	-0.539	0.527	-0.539	-0.939**	-0.345	-0.745*
A. cygnoides	-0.709*	-0.296	-0.733*	0.890**	0.296	0.418	-0.818**	-0.163	-0.781*
A. platyrhynchos	-0.878**	0.951**	0.309	0.963**	0.987**	0.987**	-0.975**	-0.636	-0.830**

Table S6: Spearman correlation coefficients between GC3 and π_n, π_s, π_n/π_s estimates obtained after masking CpG sites from the alignments.

Significance levels are showed with * (* p-value< 0.05, ** p-value < 0.01) before the FDR correction, and with two shades of red (if the correlation is positive) or green (if the correlation in negative) after FDR correction (light :p-value< 0.05, dark : p-value< 0.01).

Species	π_n				π_s				π_n/π_s			
	all	WS	SW	GC-conservative	all	WS	SW	GC-conservative	all	WS	SW	GC-conservative
M. mulatta	0,4	-0,8	0,6	0,1	0,9	1*	0,7	0,8	-1*	-0,9	-0,7	-0,8
H. sapiens	0,9	0,5	0,9	0	1*	0,9	0,9	0,8	-0,9	-0,4	-0,7	-0,7
G. gorilla	-0,2	0,2	0,2	0,2	-0,8	-0,9	0,1	-0,2	0,5	0,6	-0,3	0,2
P. troglodytes	-0,9	-1*	0,6	-0,7	0,9	0,3	0,9	-0,5	-1*	-1	-0,7	0,3
P. anubis	-0,3	-1*	-0,1	0,3	0,9	0,7	0,8	0,8	-1*	-1*	-0,7	-0,3
P. abelii	1*	0,4	1*	0,3	1*	0,9	1*	0,9	-0,9	-0,7	-0,4	-0,9
M. gallopavo	-0,6	-1*	0,3	-0,9	1*	1*	0,1	-0,5	-1*	-1*	0,1	-0,3
N. meleagris	1*	-0,7	1*	0,1	1*	1*	0,9	1*	-0,9	-0,9	-0,5	-1*
P. cristatus	0,9	-0,7	-0,1	-0,4	0,9	0,9	0,9	0,9	-0,9	-0,9	-0,9	-0,9
P. colchicus	-0,9	-0,9	0,1	-0,9	1*	0,7	0,6	-0,6	-1*	-0,9	-0,6	-0,8
A. cygnoides	0,8	-0,3	0,9	-0,2	1*	1	1*	0,9	-0,9	-0,9	0,3	-0,7
A. platyrhynchos	0,9	0	0,9	0,1	0,9	0,9	0,9	0,9	-0,9	-0,9	-1*	-0,9

Table S7: Spearman correlation coefficients between GC3 and π_n, π_s, π_n/π_s for all mutations categories.

The dataset was split in five bins of increasing GC3 and equal number of SNPs.

Significance levels are showed with * (* p-value< 0.05, ** p-value < 0.01) before the FDR correction, and with two shades of red (if the correlation is positive) or green (if the correlation in negative) after FDR correction (light :p-value< 0.05, dark : p-value< 0.01).

Supplementaty Material

species	α			ω_a			ω_{na}		
	WS	SW	GC-conservative	WS	SW	GC-conservative	WS	SW	GC-conservative
M. mulatta	0.672*	0.0181	0.381	0.090	-0.151	0.006	-0.939**	-0.466	-0.587
H. sapiens	0.515	0.878**	0.393	-0.042	0.878*	0.2	-0.6	-0.709*	-0.393
G. gorilla	0.716*	0.284	0.384	0.533	0.284	0.266	-0.833**	-0.503	-0.433
P. troglodytes	0.454	0.563	-0.212	0.127	0.696*	-0.393	-0.587	-0.430	0.187
P. anubis	0.115	-0.163	0.139	-0.418	-0.163	-0.2	-0.6	-0.103	-0.139
P. abelii	0.066	0.684*	0.272	-0.284	0.721*	0.103	-0.503	-0.660*	-0.503
M. gallopavo	0.684*	-0.078	0.284	0.321	-0.151	0.127	-0.818**	0.224	-0.345
N. meleagris	0.648*	0.624	0.624	-0.163	0.563	0.466	-0.903**	-0.721*	-0.721*
P. cristatus	-0.066	0.393	0.078	-0.187	0.393	-0.248	-0.563	-0.454	-0.345
P. colchicus	0.833*	0.030	0.757*	0.833*	-0.030	0.745*	-0.952**	-0.054	-0.721*
A. cygnoides	0.757*	-0.212	0.139	0.357	-0.236	-0.503	-0.878**	0.296	-0.284
A. platyrhynchos	0.612	0.430	-0.187	-0.903**	0.430	-0.733*	-0.963**	-0.503	-0.563

Table S8: Spearman correlation coefficients between GC3 and α, ω_a and ω_{na} estimates for the "GammaExpo" model.

Significance levels are showed with * (* p-value< 0.05, ** p-value < 0.01) before the FDR correction, and with two shades of red (if the correlation is positive) or green (if the correlation in negative) after FDR correction (light :p-value< 0.05, dark : p-value< 0.01).

Species	α				ω_a				ω_{na}			
	all	WS	SW	GC-conservative	all	WS	SW	GC-conservative	all	WS	SW	GC-conservative
M. mulatta	0,6	0,6	-0,5	0,7	-0,4	-0,6	-0,9	0,7	-1*	0,9	-0,5	-0,9
H. sapiens	0,5	0,3	0,9	0,6	0,5	-0,4	0,9	0,6	-0,7	-0,9	-0,7	-0,7
G. gorilla	-0,6	-0,8	0,6	-0,9	-0,9	-0,8	0,6	-0,9	0,2	0	-0,6	0,1
P. troglodytes	1*	0,9	1*	0,2	1*	0,5	1*	-0,6	-1*	-1*	-0,7	-0,2
P. anubis	0,7	0,9	-0,3	0,8	-0,1	0,3	-0,6	-0,1	-1*	-0,9	-0,6	-0,8
P. abelii	0,1	0,2	0,2	0,7	-0,5	-0,7	-0,1	0,6	-0,7	-0,7	-0,7	-0,7
M. gallopavo	0,97**	0,56	-0,2	0,71	0,6	-0,2	-0,2	0	-0,97**	-0,67	0,3	-0,7
N. meleagris	-0,44	0,15	0	0	-0,7	-0,4	0,3	-0,7	0,44	-0,15	0	0
P. cristatus	0,9	0,15	0,9	0,4	0,9	0,1	0,9	0,1	-0,9	-0,15	-0,9	-0,7
P. colchicus	0,7	0,2	-0,4	0,7	0	-0,5	0	0,4	-1*	-0,4	-0,3	-0,9
A. cygnoides	0,67	0,22	0,2	0,33	0,7	-0,3	0,2	-0,1	-0,97**	-0,22	0,4	-0,33
A. platyrhynchos	1*	1*	0,66	1*	-0,3	-0,8	0,7	0,1	-0,8	-1*	-0,87	-0,9

Table S9: Spearman correlation coefficients between GC3 and α, ω_a and ω_{na} estimates for the "GammaZero" model.

The dataset was split in five bins of increasing GC3 and equal number of SNPs.

Significance levels are showed with * (* p-value< 0.05, ** p-value < 0.01) before the FDR correction, and with two shades of red (if the correlation is positive) or green (if the correlation in negative) after FDR correction (light :p-value< 0.05, dark : p-value< 0.01).

Supplementaty Material

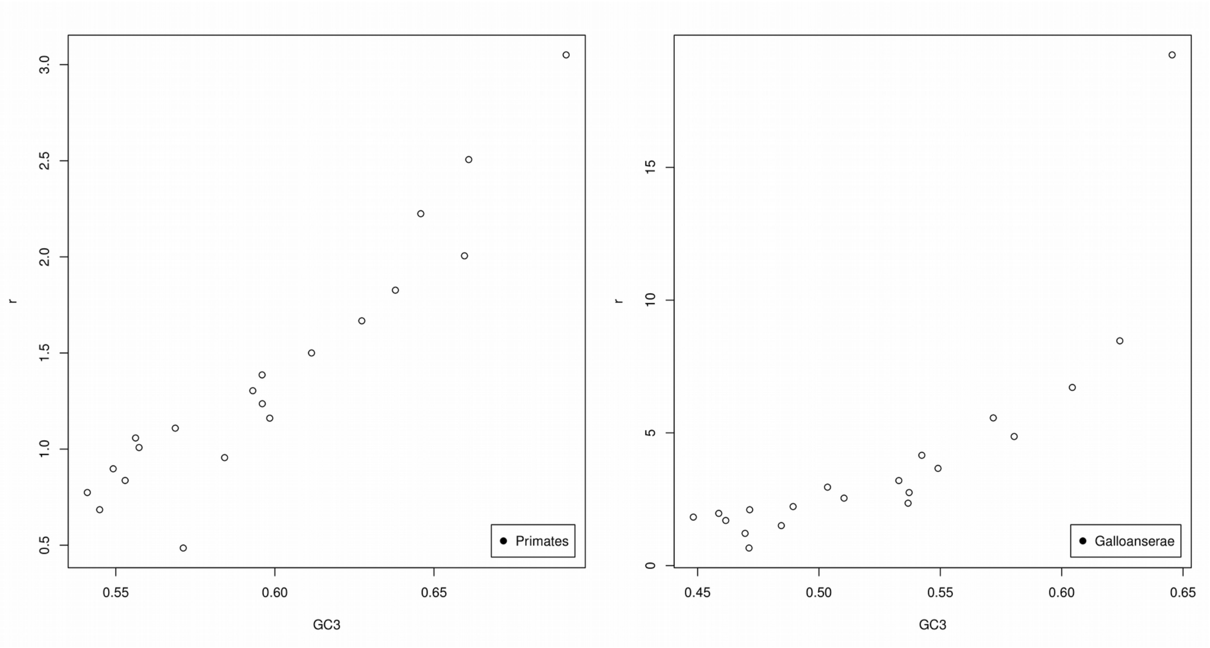

Figure S1: Correlation between GC3 and r obtained with Spearman correlation for *H. sapiens* (left) and *G. gallus* (right).

Supplementaty Material

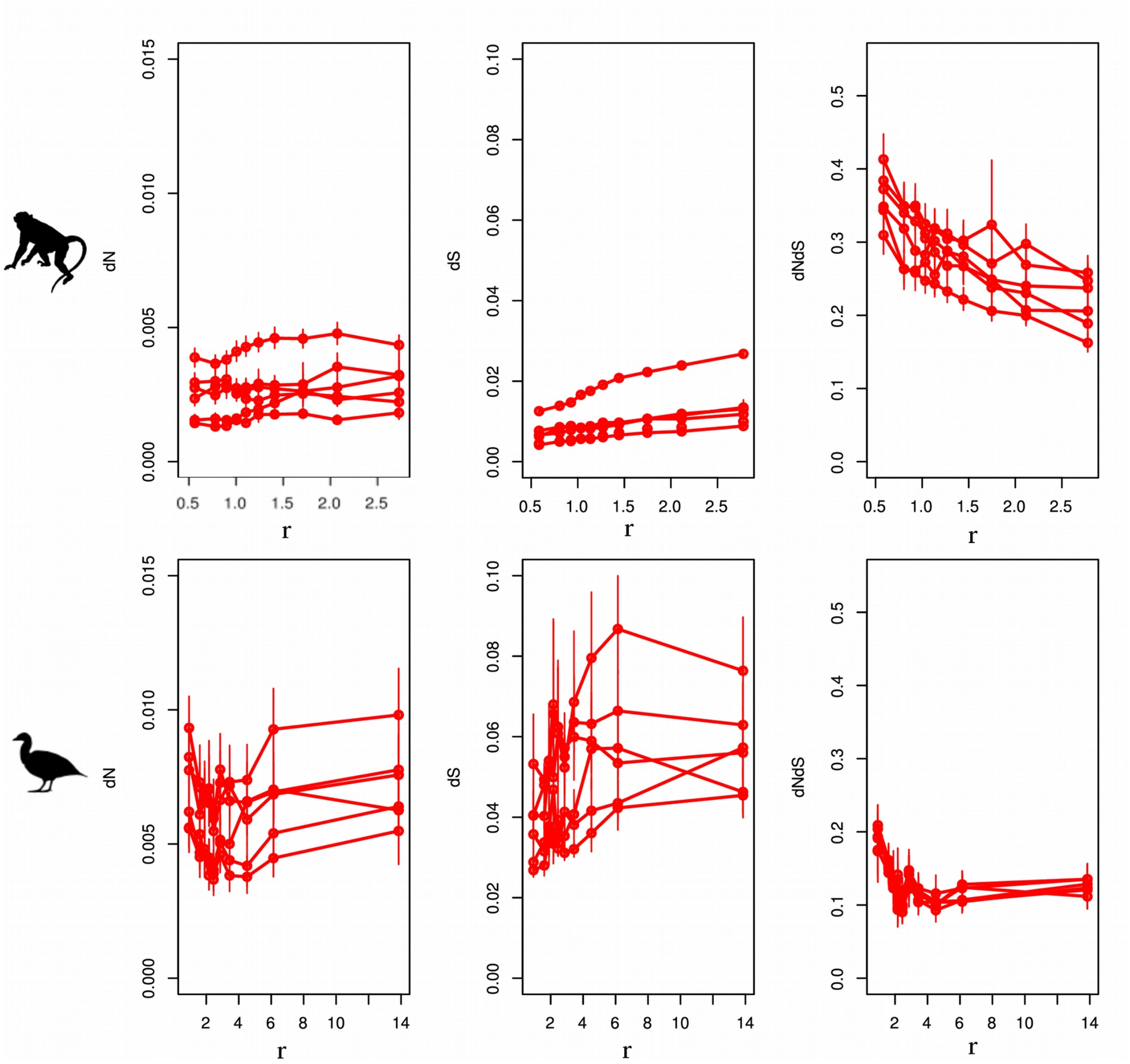

Figure S2: dN, dS and dN/dS ratio against r for each species for all substitution type taken together.

Statistics are estimated under a model assuming base composition non-stationarity (above: primates, below: fowls).

Supplementaty Material

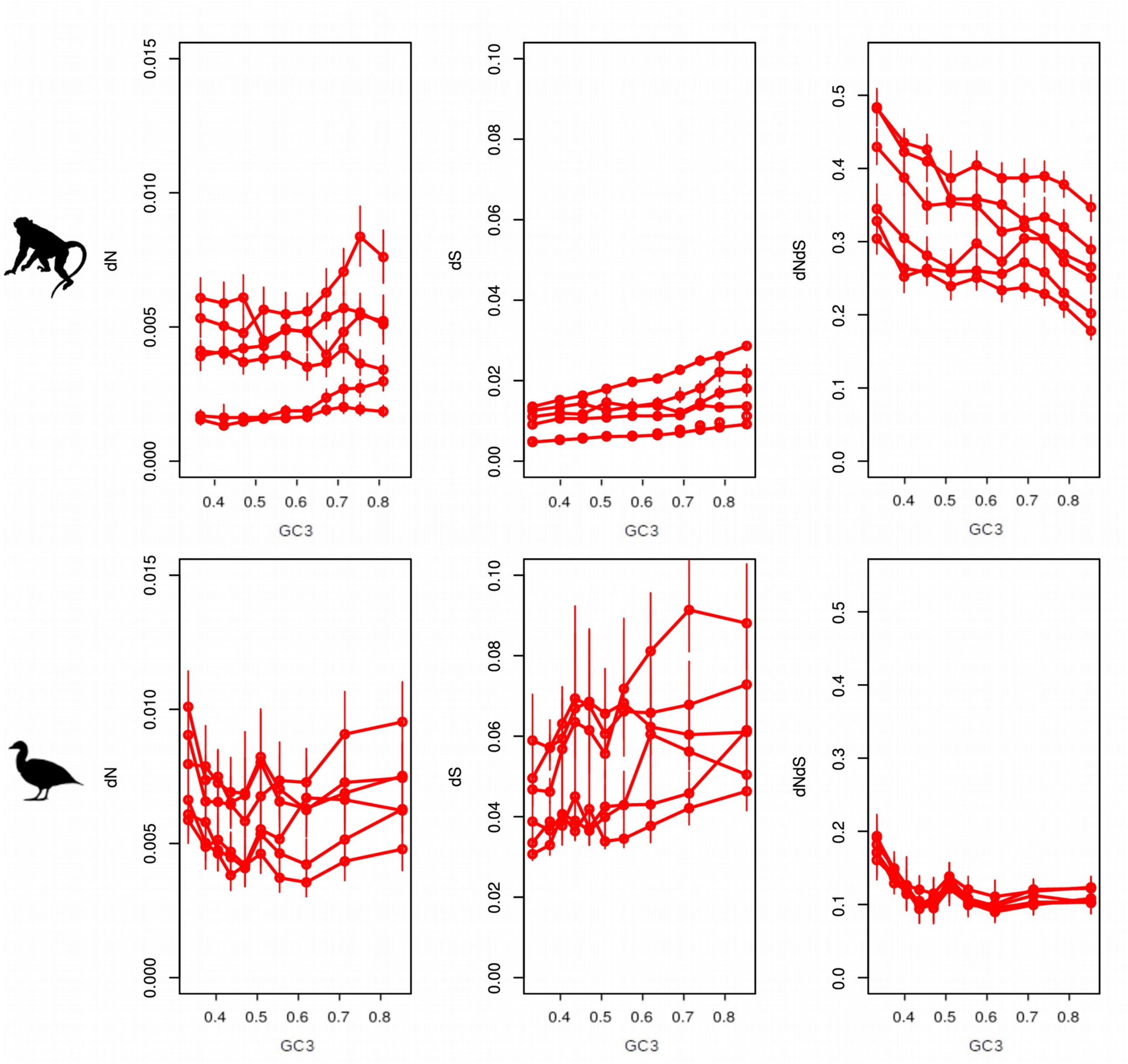

Figure S3: dN, dS and dN/dS ratio against GC3 for each species for all substitution type taken together.

Statistics are estimated under a model assuming base composition stationarity (above: primates, below: fowls).

Supplementaty Material

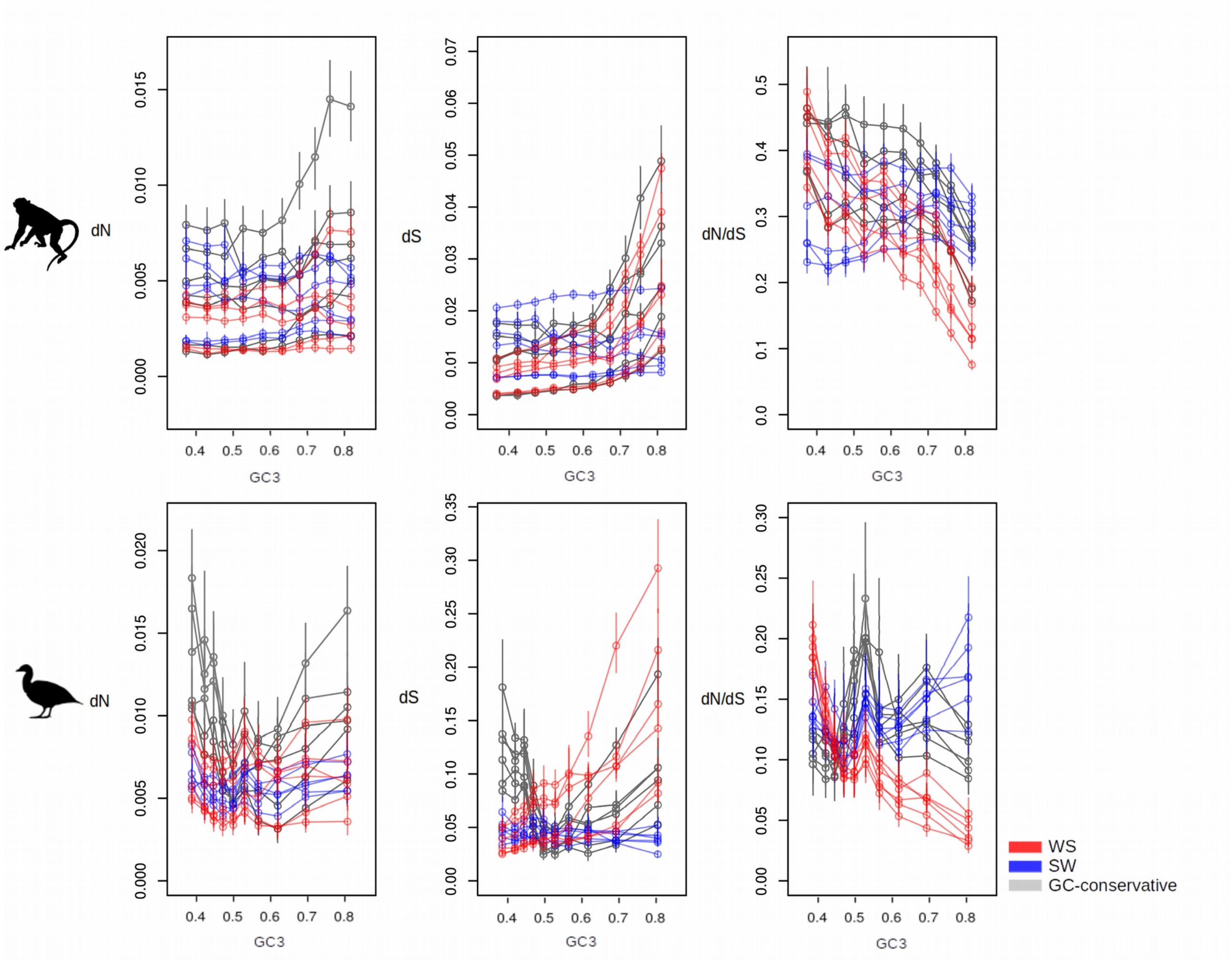

Figure S4: dN, dS and dN/dS ratio against GC3 for each species and each type of substitutions (WS, SW and GC-conservative substitution).
Statistics are estimated under a model assuming base composition stationarity (above: primates, below: fowls).

Supplementaty Material

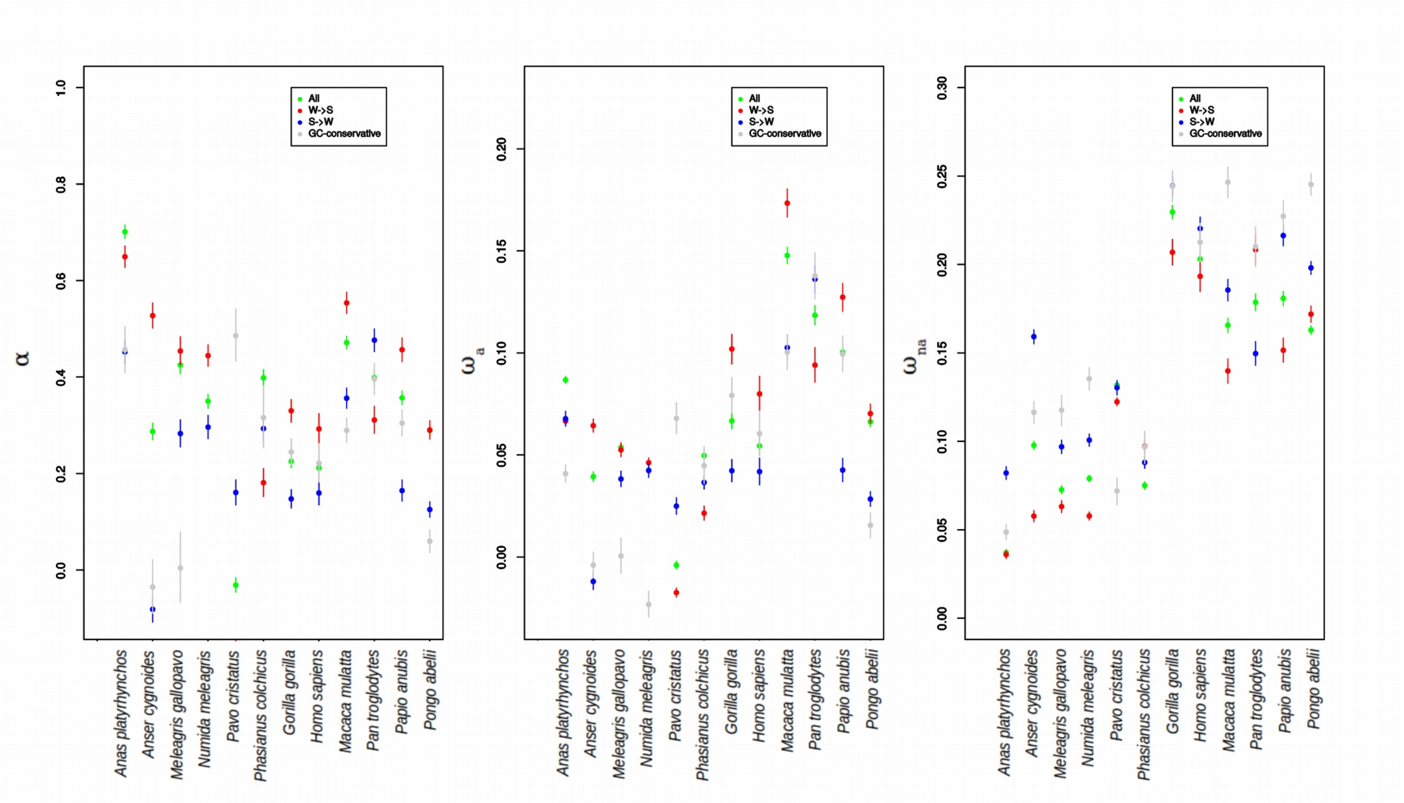

Figure S5: α, ω_a and ω_{na} estimates for each species and each type of mutations (all mutations, W→S, S→W and GC-conservative) using all genes.

Statistics are obtained using the model “GammaExpo”.

Supplementaty Material

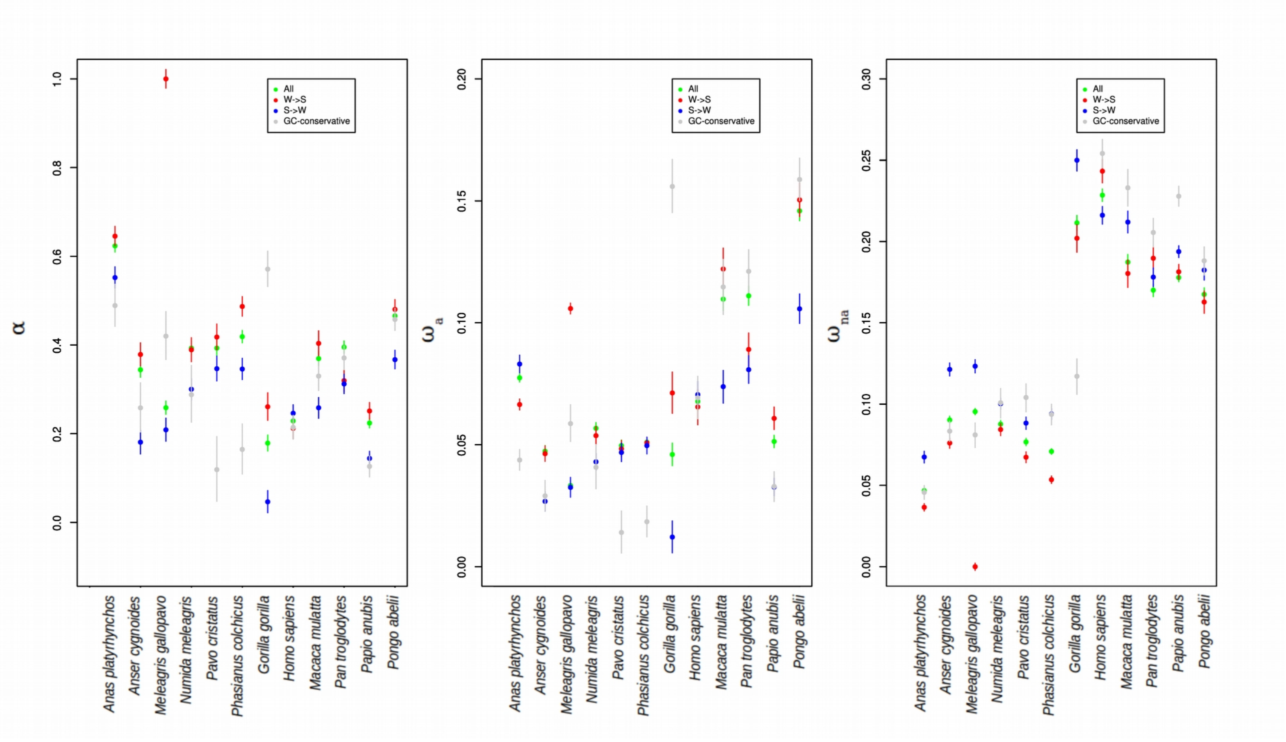

Figure S6: α, ω_a and ω_{na} estimates for each species and each type of mutations (all mutations, W→S, S→W and GC-conservative) using all genes and using only five randomly chosen individuals of each species.

Statistics are obtained using the model "GammaZero".

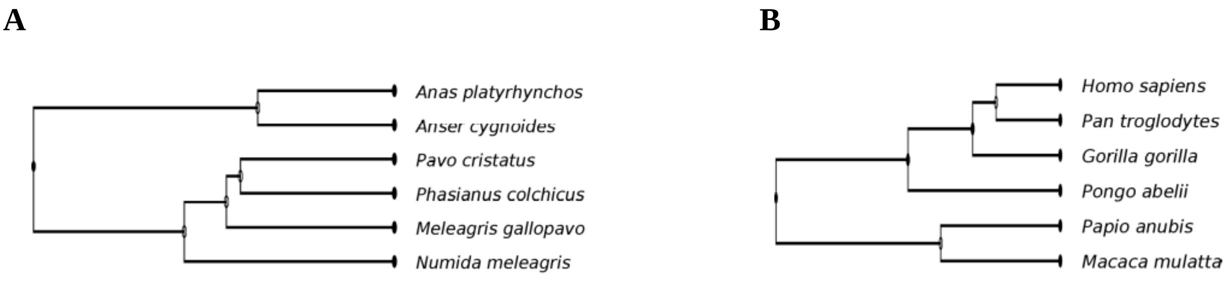

Figure S7: Tree topologies used to map substitution in Galloanserae (A) and primates (B).

Supplementary Material

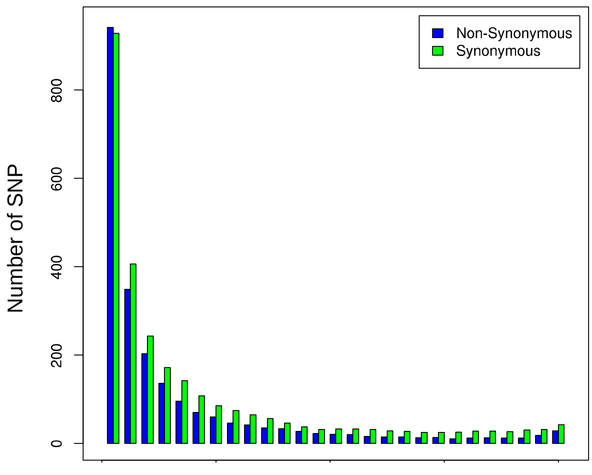

Figure S8 : Site frequency spectra of *Homo sapiens* with all mutation type without masking CpG sites.

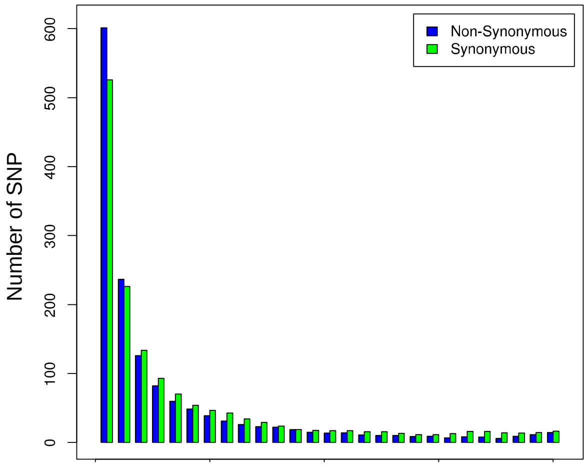

Figure S9 : Site frequency spectra of *Homo sapiens* with all mutation type with a masking of CpG sites.

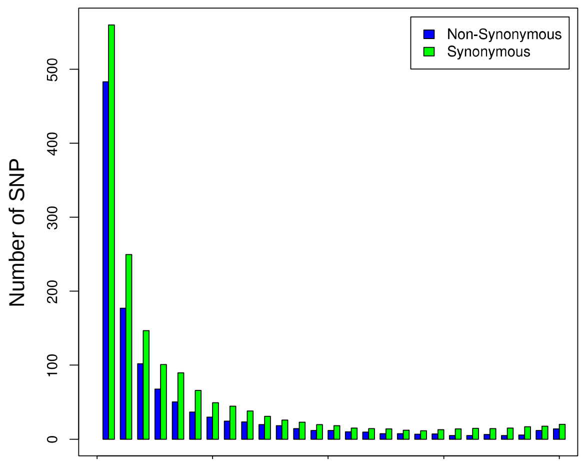

Figure S10 : Site frequency spectra of *Homo sapiens* with SW mutation type without masking CpG sites.

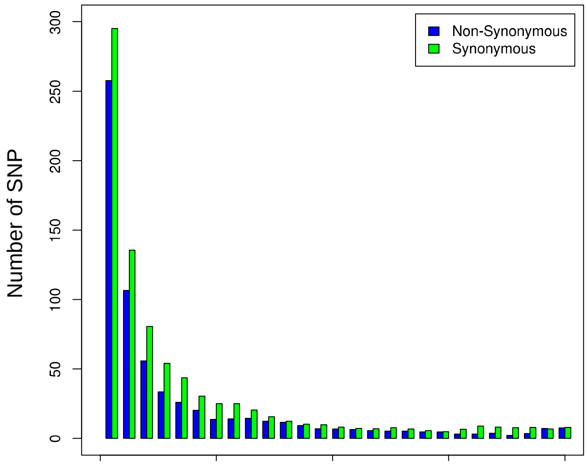

Figure S11 : Site frequency spectra of *Homo sapiens* with SW mutation type with a masking of CpG sites.

Supplementary Material

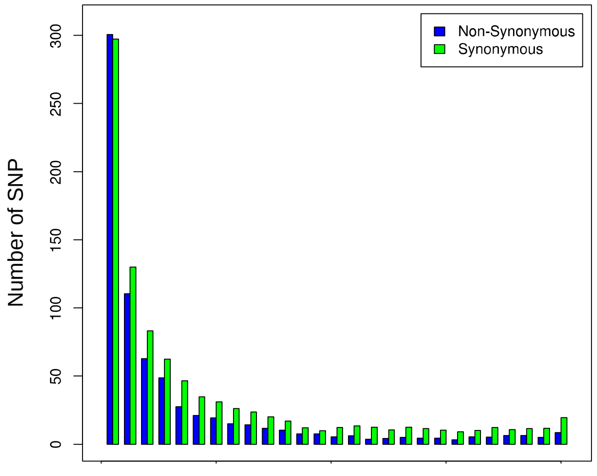

Figure S12 : Site frequency spectra of *Homo sapiens* with WS mutations without masking CpG sites.

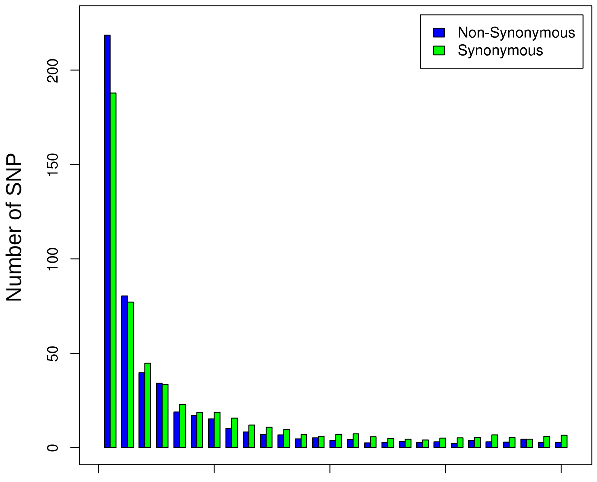

Figure S13 : Site frequency spectra of *Homo sapiens* with WS mutations with a masking of CpG sites.

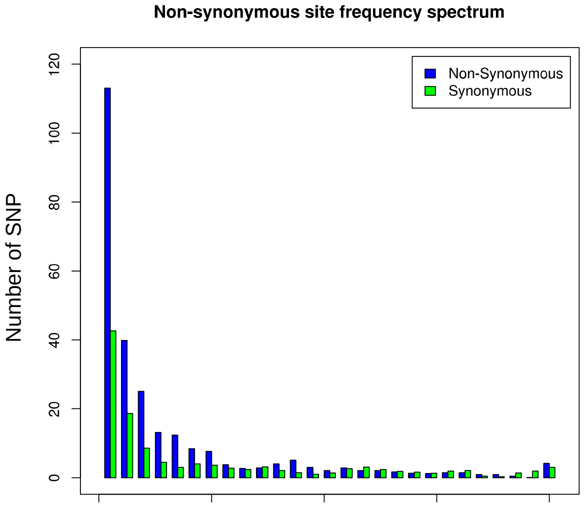

Figure S14 : Site frequency spectra of *Homo sapiens* with GC-conservative mutations without masking CpG sites.

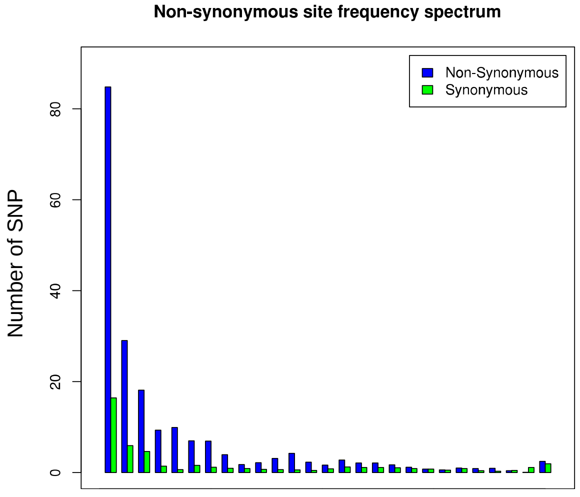

Figure S15 : Site frequency spectra of *Homo sapiens* with GC-conservative mutations with a masking of CpG sites.

Supplementary Material

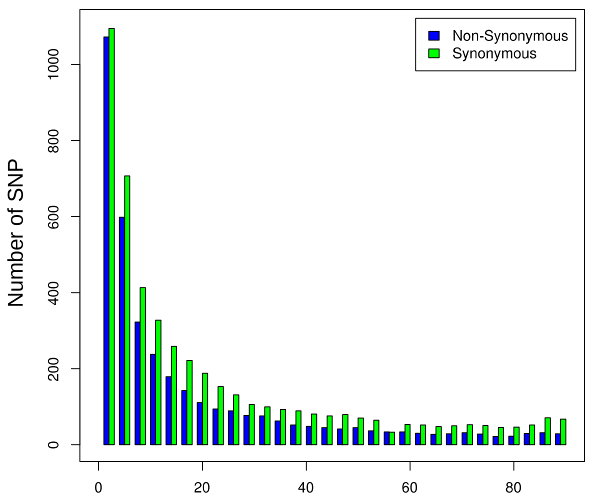

Figure S16 : Site frequency spectra of *Gorilla gorilla* with all mutation type without masking CpG sites.

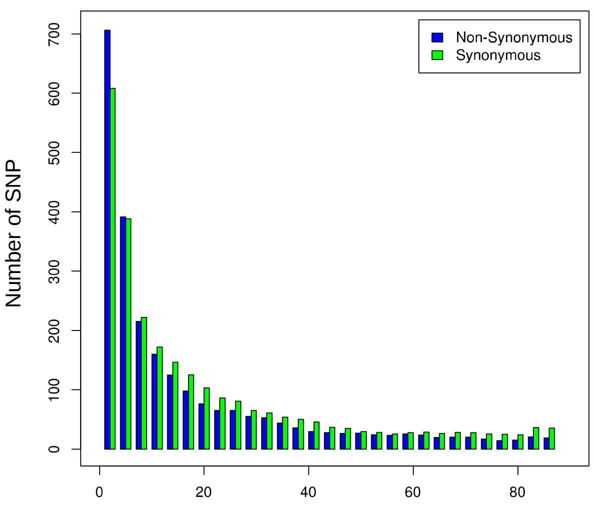

Figure S17 : Site frequency spectra of *Gorilla gorilla* with all mutation type with a masking of CpG sites.

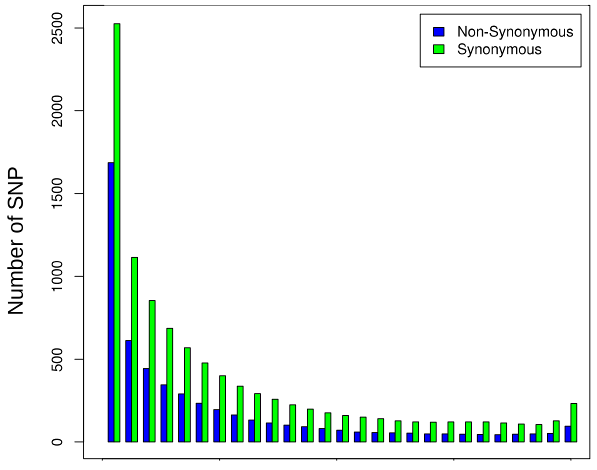

Figure S18 : Site frequency spectra of *Macaca mulatta* with all mutation type without masking CpG sites.

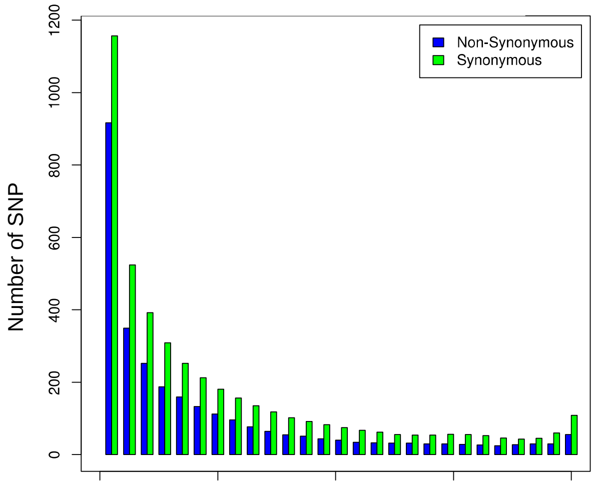

Figure S19 : Site frequency spectra of *Macaca mulatta* with all mutation type with a masking of CpG sites.

Supplementary Material

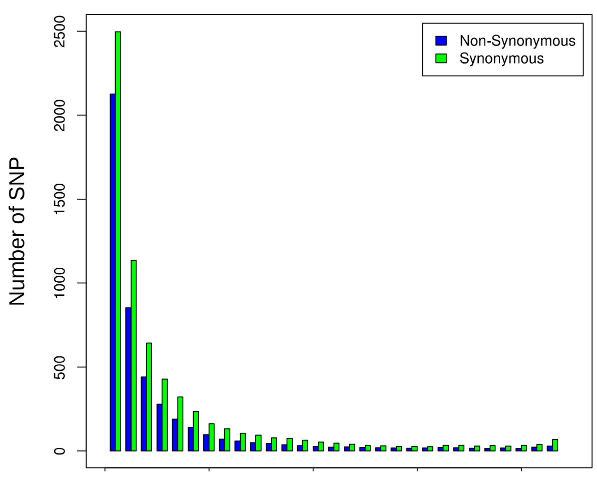

Figure S20 : Site frequency spectra of *Pan troglodytes* with all mutation type without masking CpG sites.

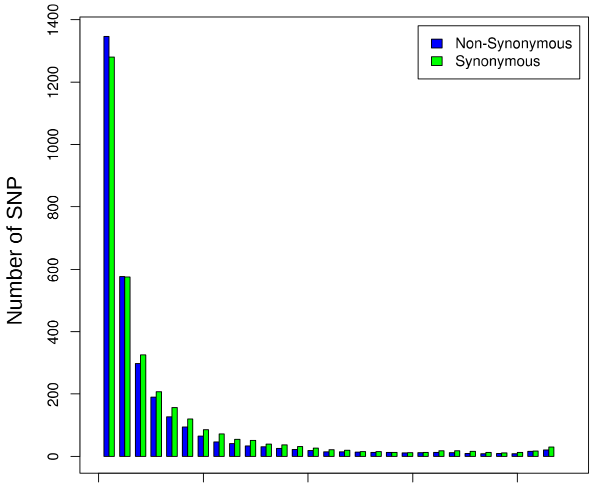

Figure S21 : Site frequency spectra of *Pan troglodytes* with all mutation type with a masking of CpG sites.

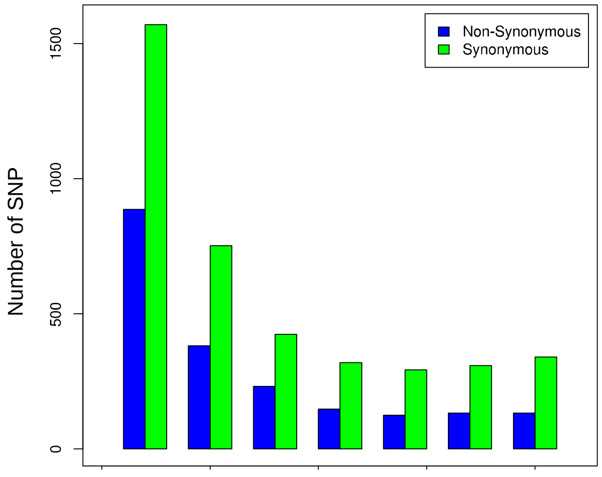

Figure S22 : Site frequency spectra of *Papio anubis* with all mutation type without masking CpG sites.

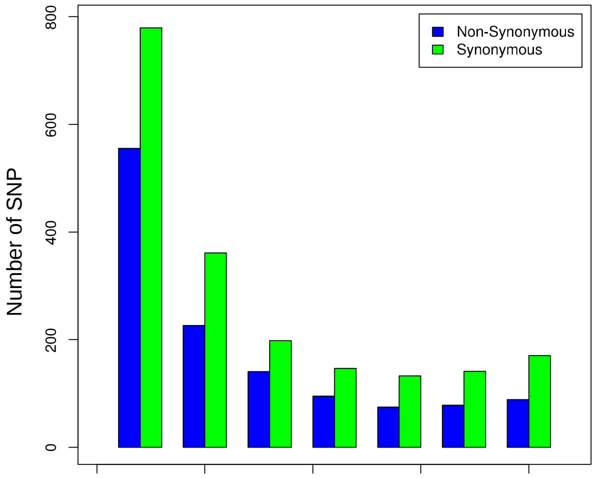

Figure S23 : Site frequency spectra of *Papio anubis* with all mutation type with a masking of CpG sites.

Supplementary Material

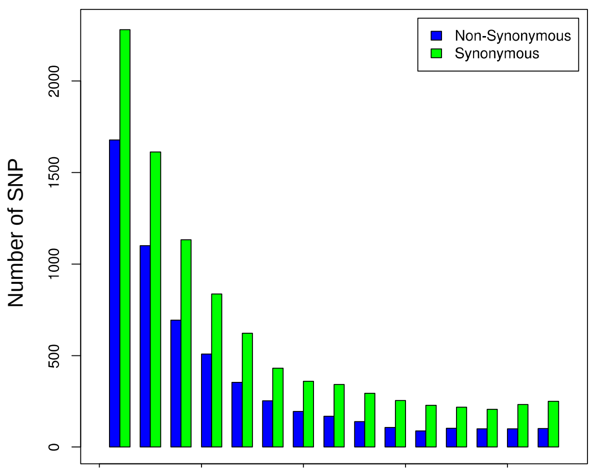

Figure S24 : Site frequency spectra of *Pongo abelii* with all mutation type without masking CpG sites.

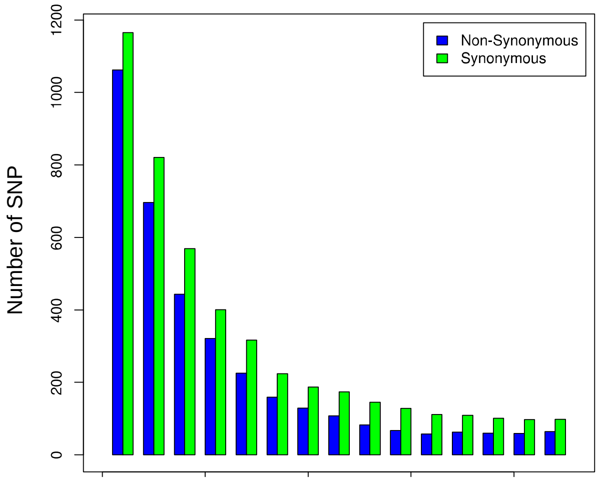

Figure S25 : Site frequency spectra of *Pongo abelii* with all mutation type with a masking of CpG sites.

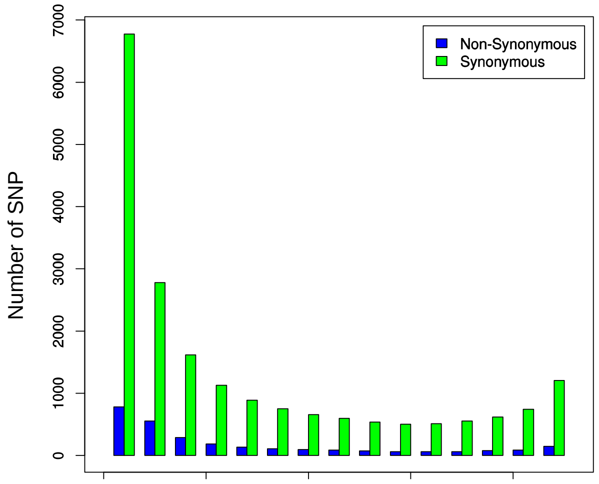

Figure S26 : Site frequency spectra of *Anas platyrhynchos* with all mutation type without masking CpG sites.

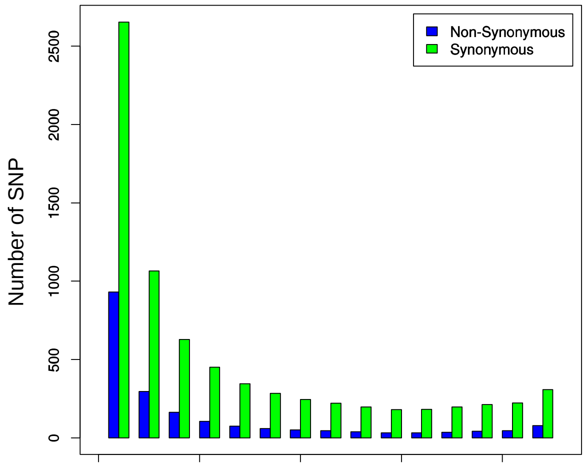

Figure S27 : Site frequency spectra of *Anas platyrhynchos* with all mutation type with a masking of CpG sites.

Supplementary Material

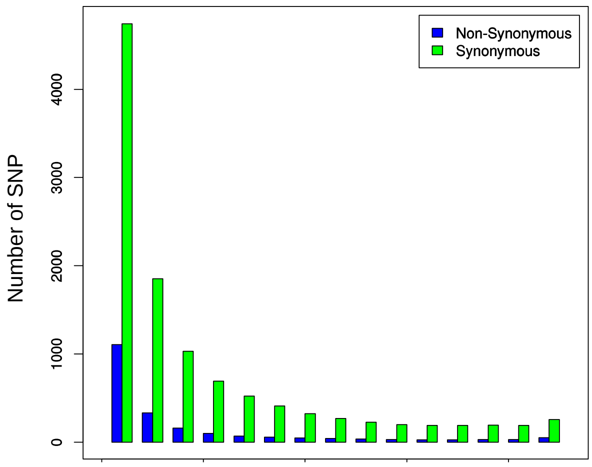

Figure S28 : Site frequency spectra of *Anas platyrhynchos* with SW mutations without masking CpG sites.

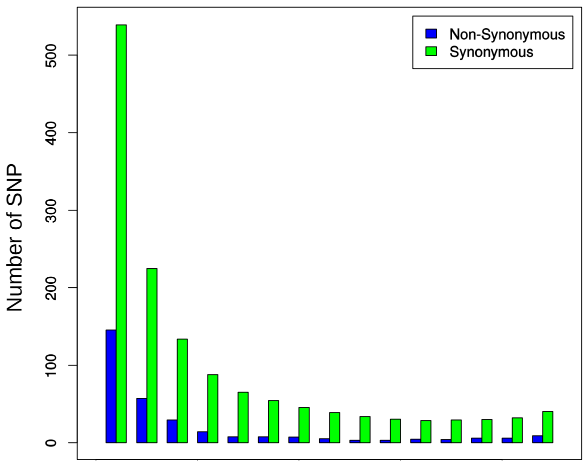

Figure S29 : Site frequency spectra of *Anas platyrhynchos* with SW mutations with a masking of CpG sites.

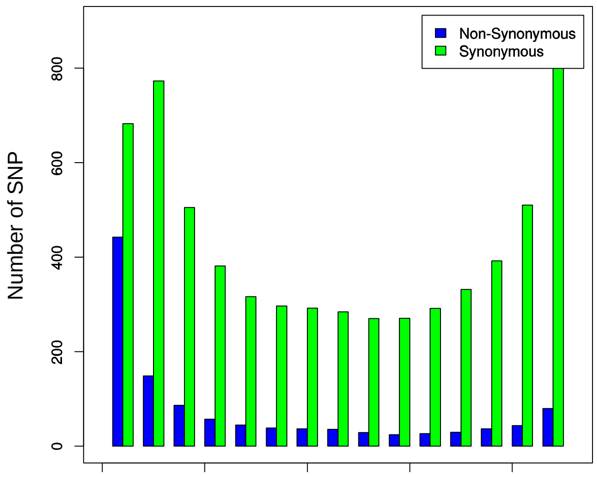

Figure S30 : Site frequency spectra of *Anas platyrhynchos* with WS mutations without masking CpG sites.

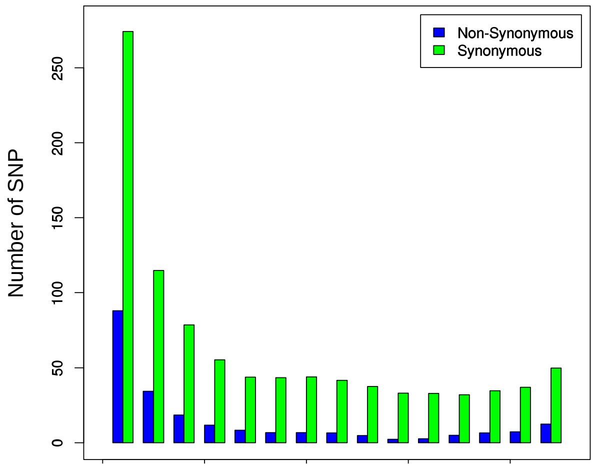

Figure S31 : Site frequency spectra of *Anas platyrhynchos* with WS mutations with a masking of CpG sites.

Supplementary Material

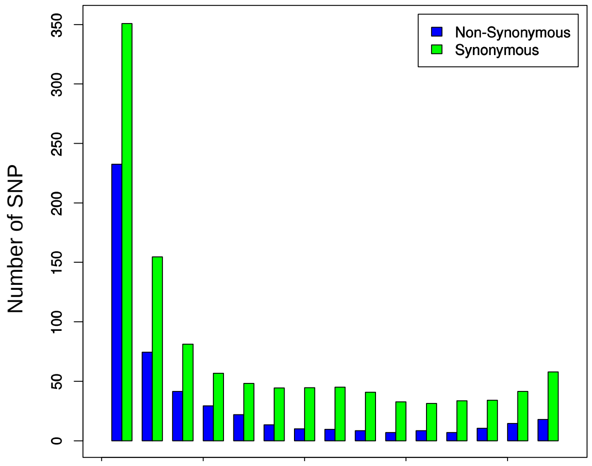

Figure S32 : Site frequency spectra of *Anas platyrhynchos* with GC-conservative mutations without masking CpG sites.

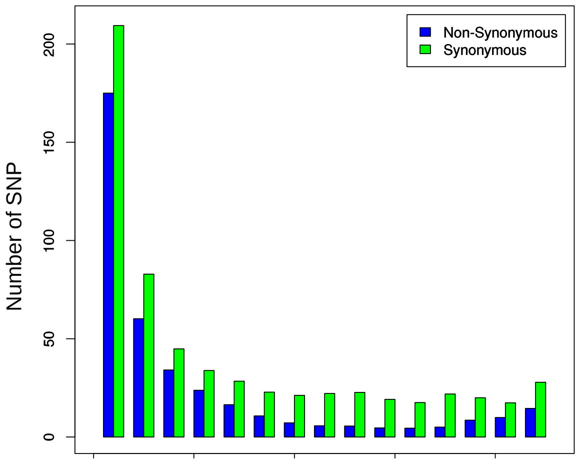

Figure S33 : Site frequency spectra of *Anas platyrhynchos* with GC-conservative mutations with a masking of CpG sites.

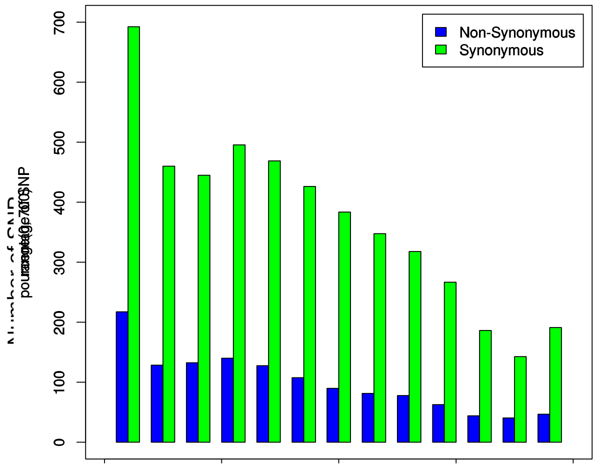

Figure S34 : Site frequency spectra of *Anser cygnoides* with all mutation type without masking CpG sites.

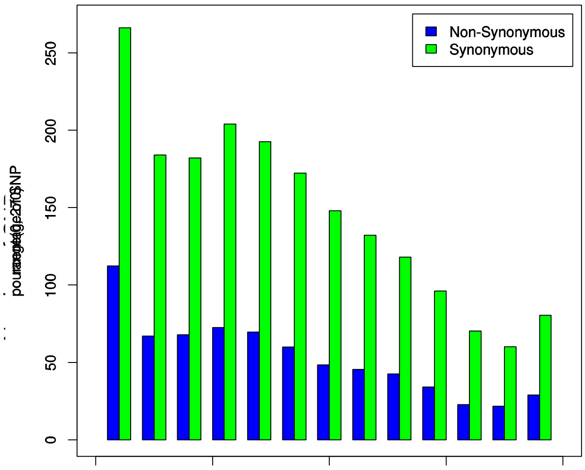

Figure S35 : Site frequency spectra of *Anser cygnoides* with all mutation type with a masking of CpG sites.

Supplementary Material

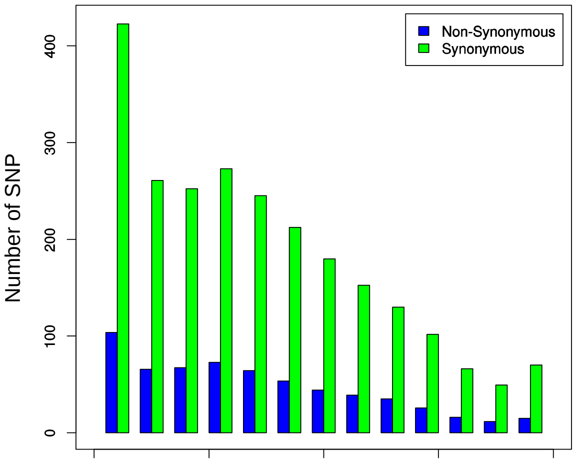

Figure S36 : Site frequency spectra of *Anser cygnoides* with SW mutations without masking CpG sites.

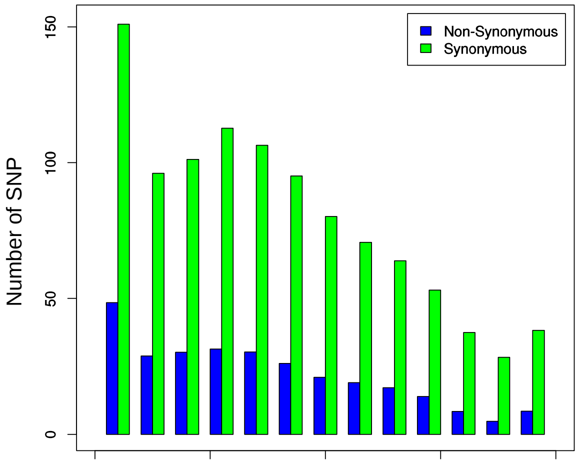

Figure S37 : Site frequency spectra of *Anser cygnoides* with SW mutations with a masking of CpG sites.

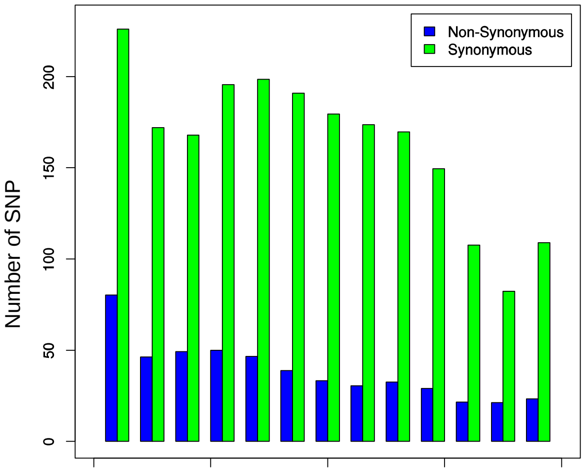

Figure S38 : Site frequency spectra of *Anser cygnoides* with WS mutations without masking CpG sites.

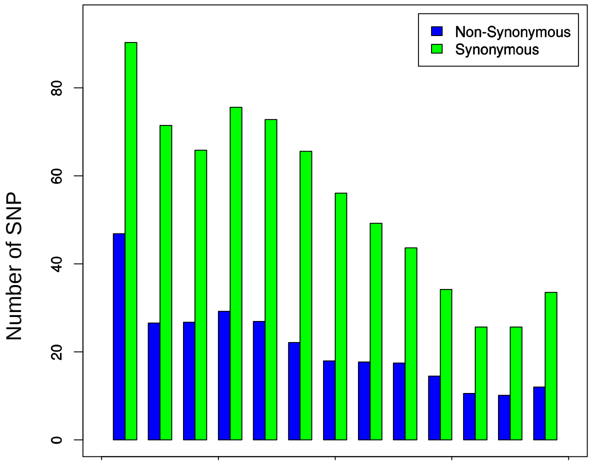

Figure S39: Site frequency spectra of *Anser cygnoides* with WS mutations with a masking of CpG sites.

Supplementary Material

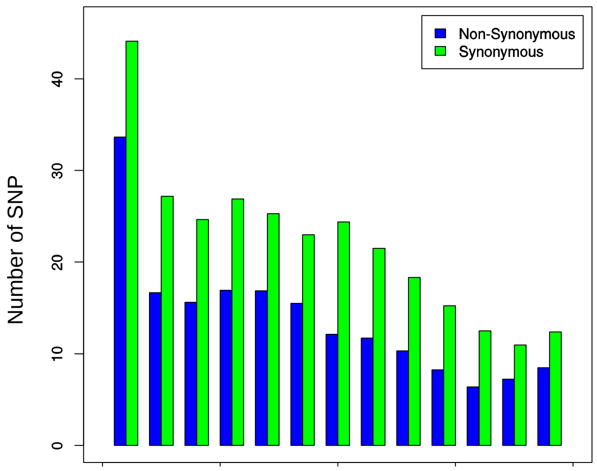

Figure S40 : Site frequency spectra of *Anser cygnoides* with GC-conservative mutations without masking CpG sites.

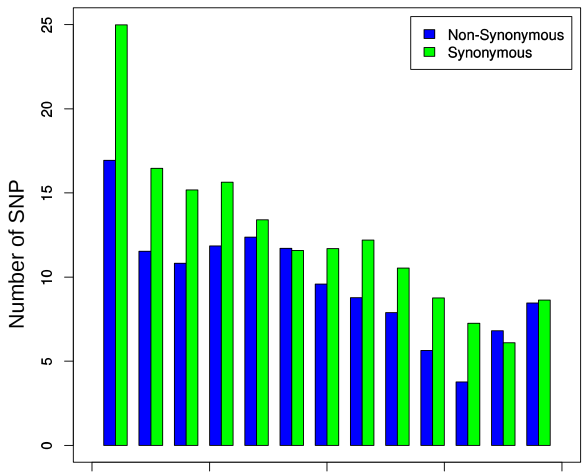

Figure S41: Site frequency spectra of *Anser cygnoides* with GC-conservative mutations with a masking of CpG sites.

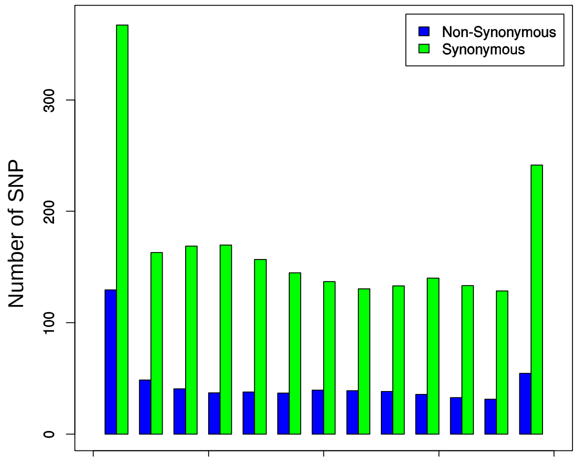

Figure S42 : Site frequency spectra of *Pavo cristatus* with all mutation type without masking CpG sites.

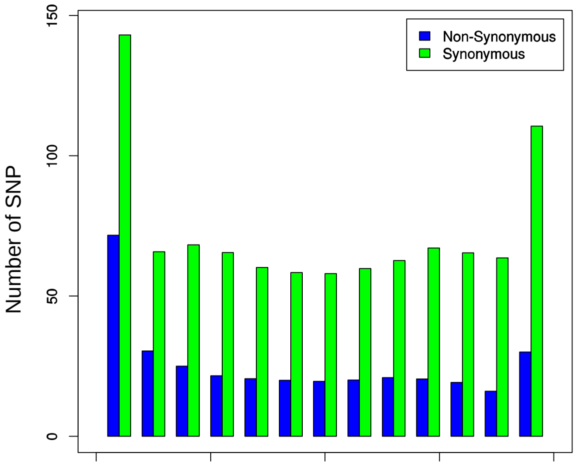

Figure S43: Site frequency spectra of *Pavo cristatus* with all mutation type with a masking of CpG sites.

Supplementary Material

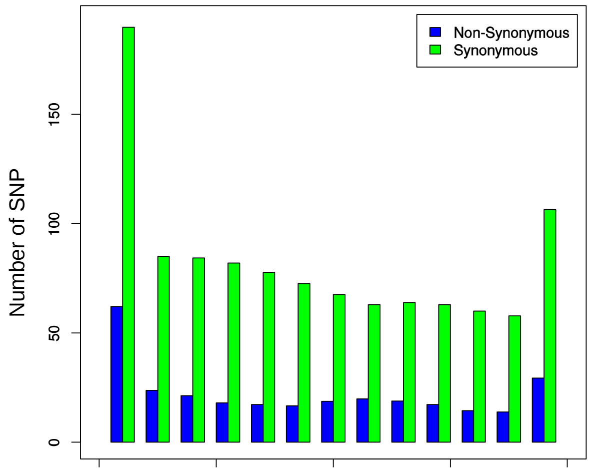

Figure S44 : Site frequency spectra of *Pavo cristatus* with SW mutations without masking CpG sites.

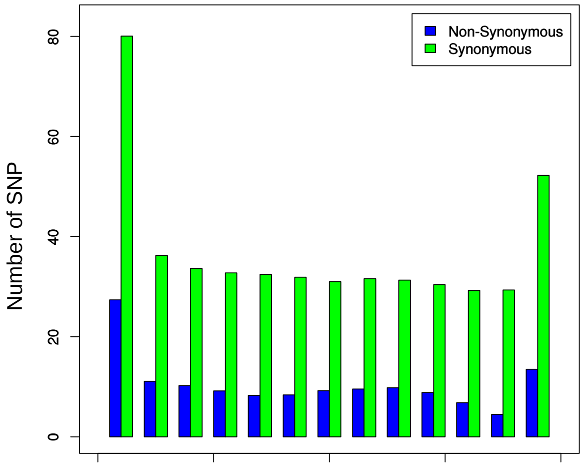

Figure S45: Site frequency spectra of *Pavo cristatus* with SW mutations with a masking of CpG sites.

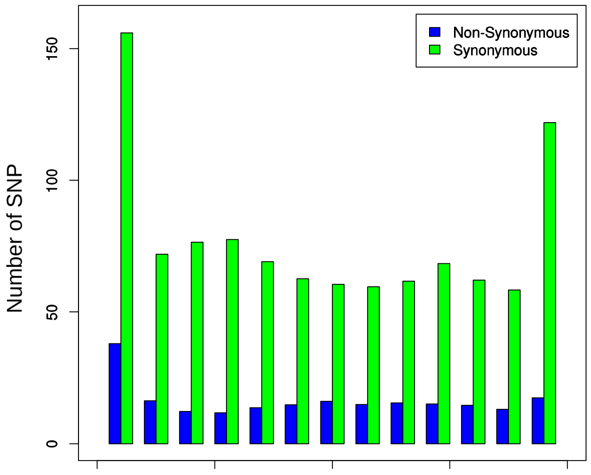

Figure S46 : Site frequency spectra of *Pavo cristatus* with WS mutations without masking CpG sites.

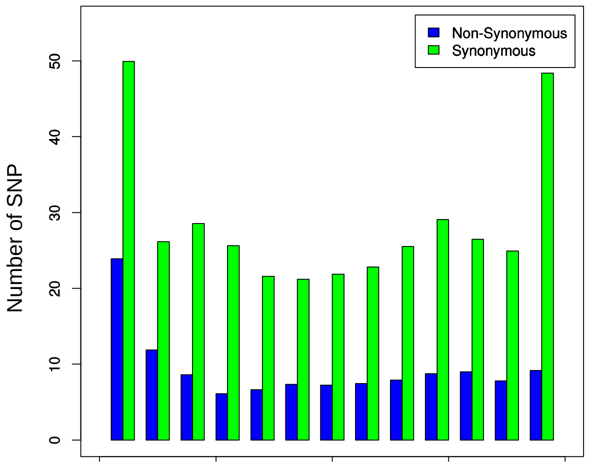

Figure S47: Site frequency spectra of *Pavo cristatus* with WS mutations with a masking of CpG sites.

Supplementary Material

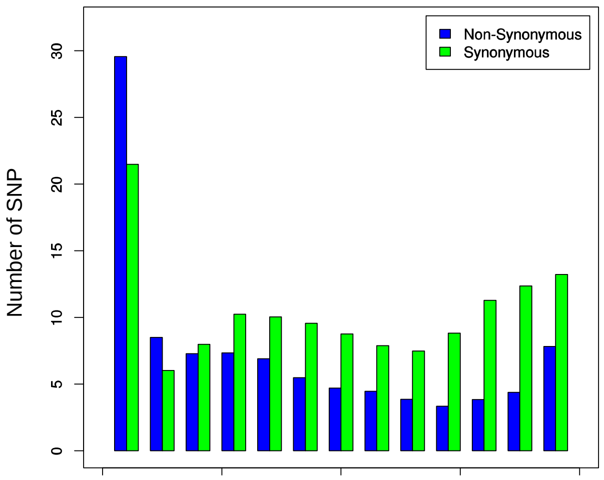

Figure S48 : Site frequency spectra of *Pavo cristatus* with GC-conservative mutations without masking CpG sites.

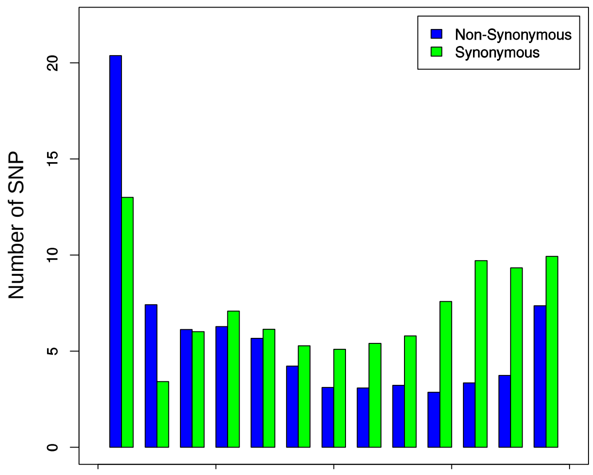

Figure S49: Site frequency spectra of *Pavo cristatus* with GC-conservative mutations with a masking of CpG sites.

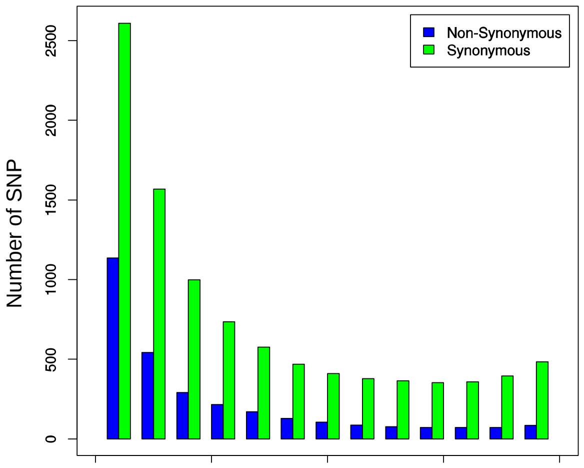

Figure S50 : Site frequency spectra of *Phasianus colchicus* with all mutation type without masking CpG sites.

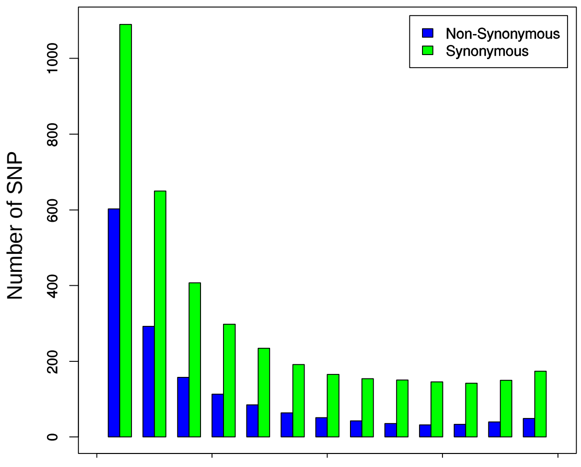

Figure S51: Site frequency spectra of *Phasianus colchicus* with all mutation type with a masking of CpG sites.

Supplementary Material

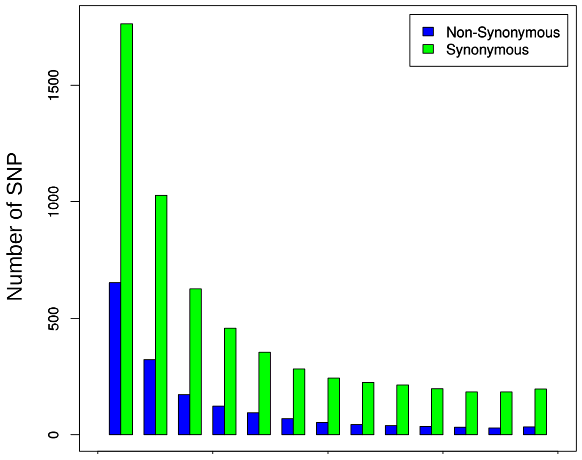

Figure S52 : Site frequency spectra of *Phasianus colchicus* with SW mutations without masking CpG sites.

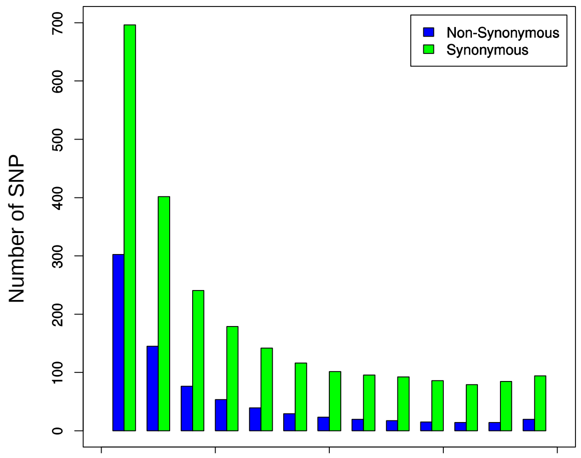

Figure S53: Site frequency spectra of *Phasianus colchicus* with SW mutations with a masking of CpG sites.

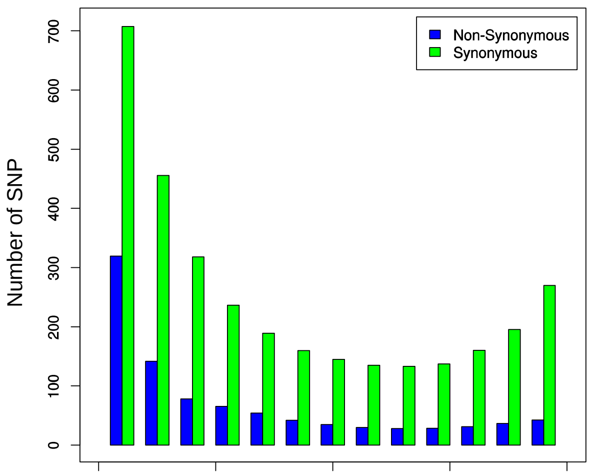

Figure S54 : Site frequency spectra of *Phasianus colchicus* with WS mutations without masking CpG sites.

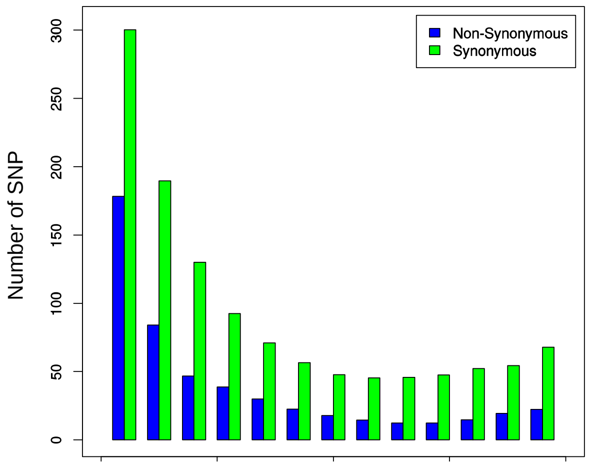

Figure S55: Site frequency spectra of *Phasianus colchicus* with WS mutations with a masking of CpG sites.

Supplementary Material

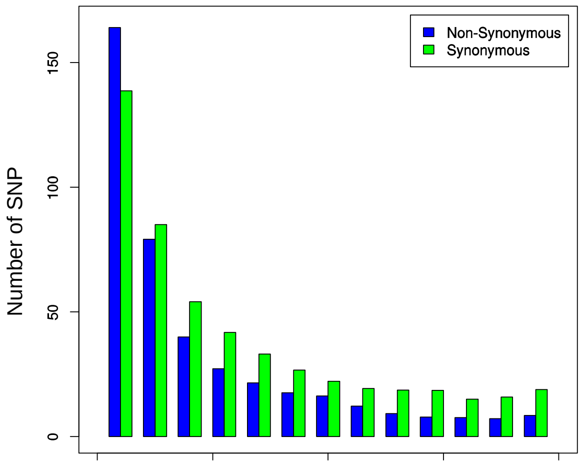

Figure S56 : Site frequency spectra of *Phasianus colchicus* with GC-conservative mutations without masking CpG sites.

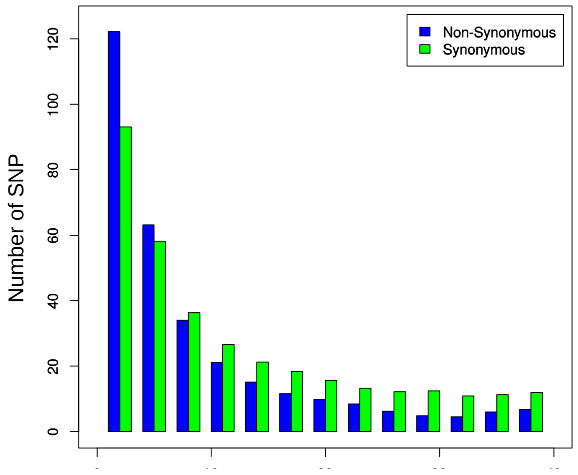

Figure S57: Site frequency spectra of *Phasianus colchicus* with GC-conservative mutations with a masking of CpG sites.

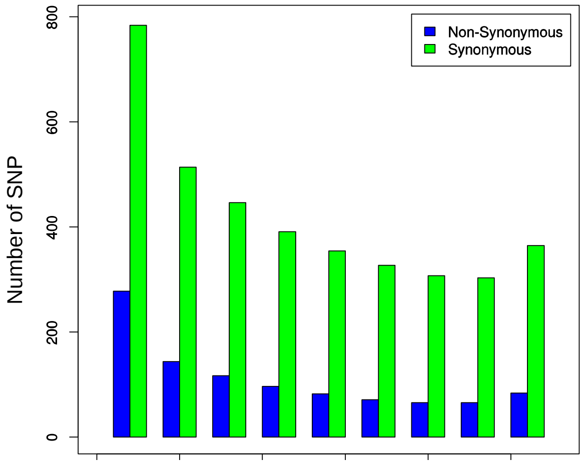

Figure S58 : Site frequency spectra of *Meleagris gallopavo* with all mutation type without masking CpG sites.

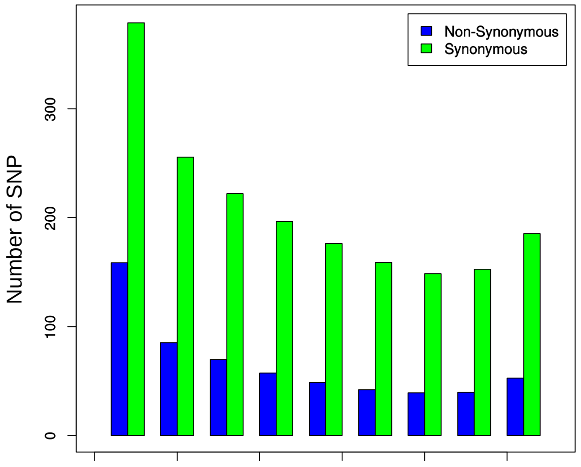

Figure S59: Site frequency spectra of *Meleagris gallopavo* with all mutation type with a masking of CpG sites.

Supplementary Material

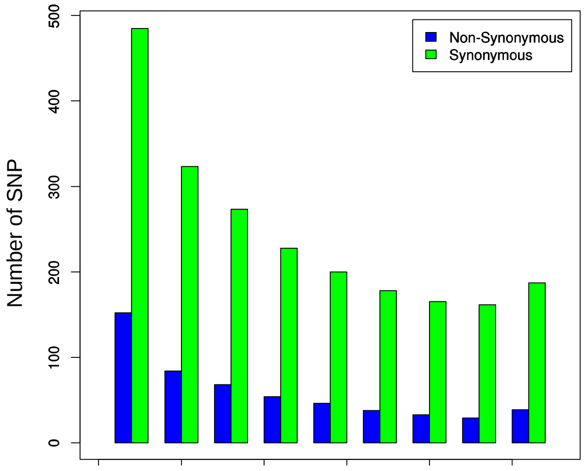

Figure S60 : Site frequency spectra of *Meleagris gallopavo* with SW mutations without masking CpG sites.

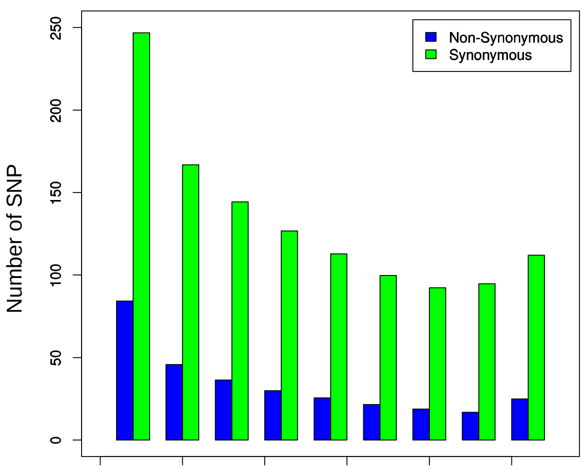

Figure S61: Site frequency spectra of *Meleagris gallopavo* with SW mutations with a masking of CpG sites.

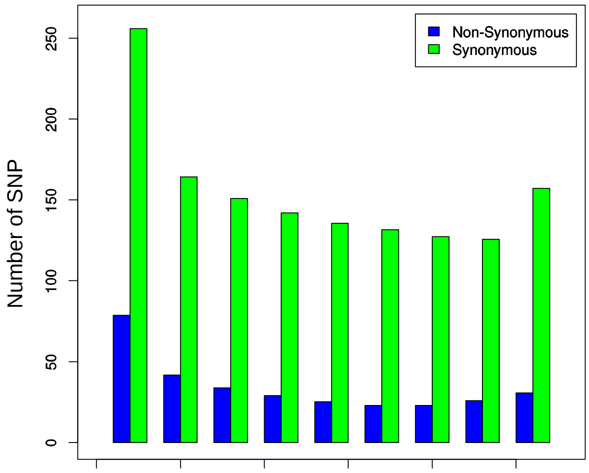

Figure S62 : Site frequency spectra of *Meleagris gallopavo* with WS mutations without masking CpG sites.

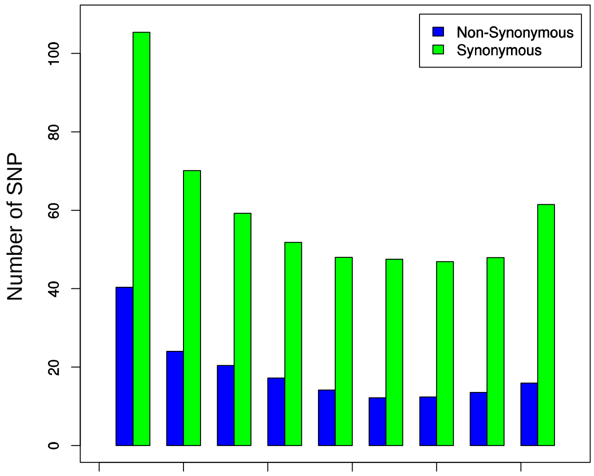

Figure S63: Site frequency spectra of *Meleagris gallopavo* with WS mutations with a masking of CpG sites.

Supplementary Material

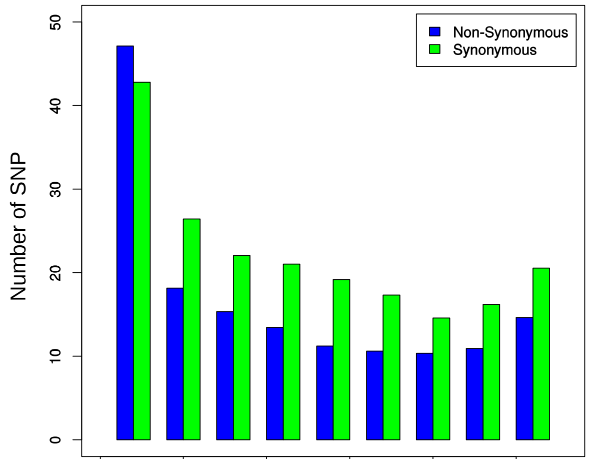

Figure S64 : Site frequency spectra of *Meleagris gallopavo* with GC-conservative mutations without masking CpG sites.

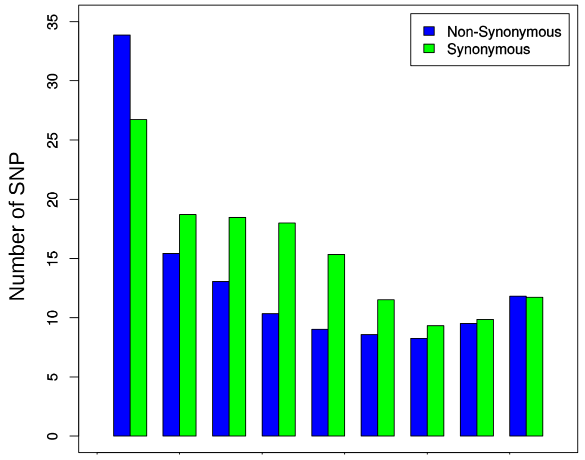

Figure S65: Site frequency spectra of *Meleagris gallopavo* with GC-conservative mutations with a masking of CpG sites.

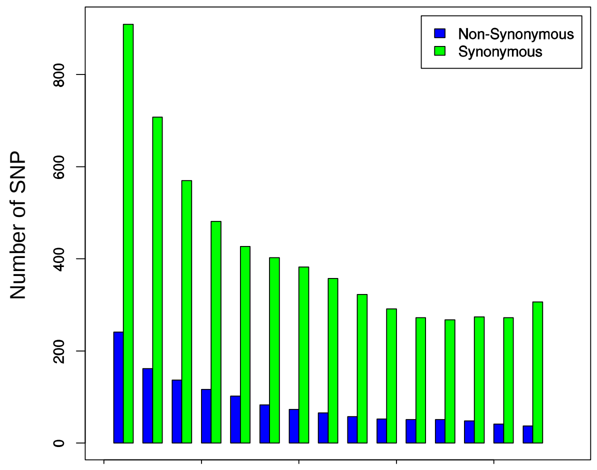

Figure S66 : Site frequency spectra of *Numida gallopavo* with all mutation type without masking CpG sites.

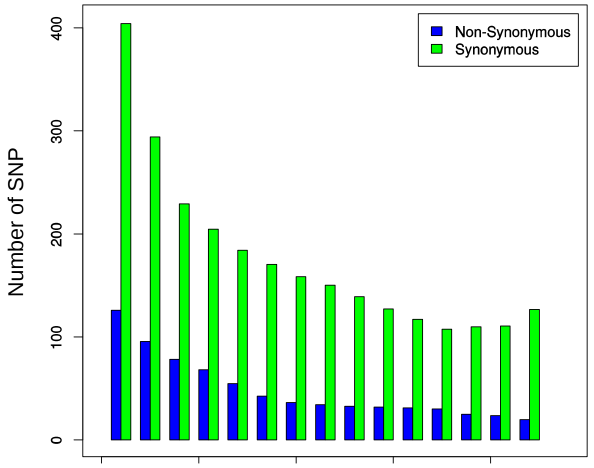

Figure S67: Site frequency spectra of *Numida meleagris* with all mutation type with a masking of CpG sites.

Supplementary Material

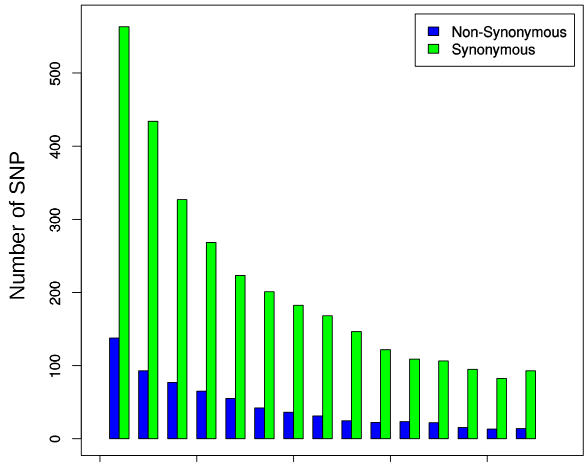

Figure S68 : Site frequency spectra of *Numida gallopavo* with SW mutations without masking CpG sites.

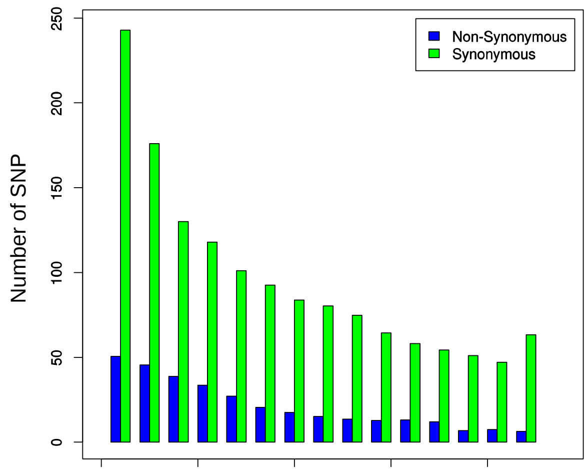

Figure S69: Site frequency spectra of *Numida meleagris* with SW mutations with a masking of CpG sites.

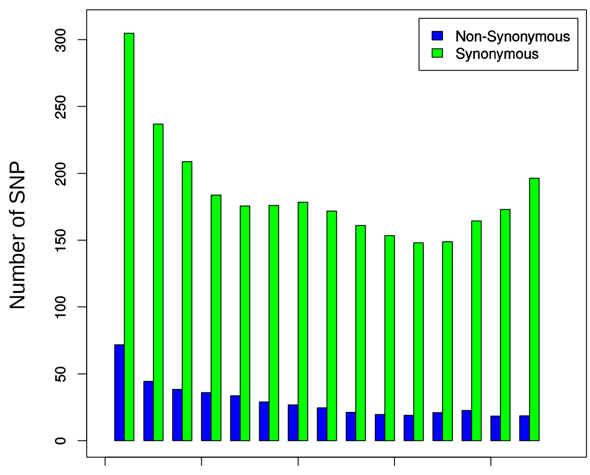

Figure S70 : Site frequency spectra of *Numida gallopavo* with WS mutations without masking CpG sites.

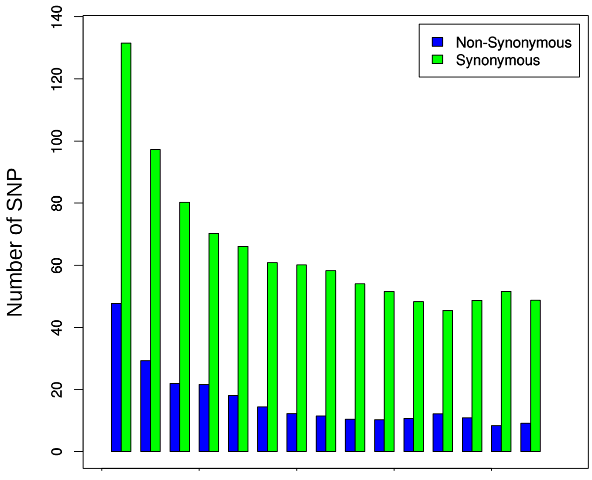

Figure S71: Site frequency spectra of *Numida meleagris* with WS mutations with a masking of CpG sites.

Supplementary Material

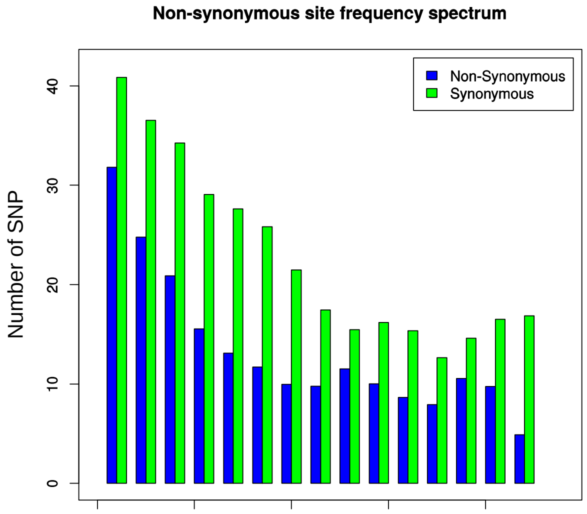

Figure S72 : Site frequency spectra of *Numida gallopavo* with GC-conservative mutations without masking CpG sites.

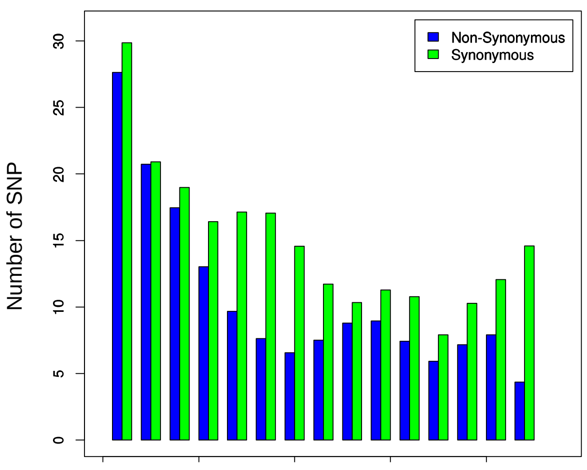

Figure S73: Site frequency spectra of *Numida meleagris* with GC-conservative mutations with a masking of CpG sites.
